# The global burden and temporal trend of cancer attributable to high body mass index: Estimates from the Global Burden of Disease Study 2019

**DOI:** 10.3389/fnut.2022.918330

**Published:** 2022-07-26

**Authors:** Xing Zhi, Xiao-hong Kuang, Kuan Liu, Jian Li

**Affiliations:** ^1^Department of General Surgery, Mianyang Central Hospital, School of Medicine, University of Electronic Science and Technology of China, Mianyang, China; ^2^Department of Hematology, The Third Hospital of Mianyang Sichuan Mental Health Center, Mianyang, China; ^3^Department of General Surgery, The Third Hospital of Mianyang Sichuan Mental Health Center, Mianyang, China

**Keywords:** cancer, high body mass index, mortality, disability-adjusted life-years, time trend

## Abstract

**Objective:**

The purpose of the study was to describe the burden and temporal trend of cancer attributable to high body mass index (BMI), with major patterns highlighted by sex, Socio-demographic Index (SDI), and geographical region.

**Methods:**

This population-based observational study collected epidemiological data on cancer attributable to high BMI from the Global Burden of Diseases (GBD) 2019. The obtained data included deaths, disability-adjusted life-years (DALYs), and their age-standardized rates at the global, gender, SDI, regional, and country levels. The trend magnitudes and directions over time for mortality were analyzed. The associations between SDI and burden of cancer attributable to high BMI were also evaluated by Pearson correlation analysis.

**Results:**

Worldwide, 462.55 thousand deaths and 11.18 million DALYs of cancer were related to high BMI in 2019, and both have more than doubled since 1990. An annual 0.6% increase was observed for the age-standardized mortality rate (ASMR), and the rate of increase slowed after 2000. In general, the burden of cancer attributable to high BMI was heavier in regions with higher SDI levels, whereas the increase slowed down or even showed a decreasing trend in the recent years. In contrast, in regions with lower SDI levels, although the baseline burden of cancer attributable to high BMI was relatively low, both the numbers and rates of deaths and DALYs showed a significantly increasing trend and may not stop increasing for a period of time. The trend and magnitude of high BMI-related cancer burden varied substantially in different anatomical sites. The leading three cancers of DALYs attributable to high BMI in 2019 were esophageal, colorectal, and liver cancer.

**Conclusion:**

The high BMI-related burden of cancers is worsening, particularly in developing countries. Concerted action should be suggested to increase awareness of the harmful effects of high BMI and decrease the burden of disease attributable to high BMI, including cancer.

## Introduction

Excess body weight, especially obesity, is the number one lifestyle-related risk factor for premature death, which increases the incidence and mortality of various diseases and conditions, including cardiovascular disease (CVD), type 2 diabetes mellitus (T2DM), non-alcoholic fatty liver disease (NAFLD), and certain types of cancer (1). Following CVD and T2DM, cancer is the third leading cause of mortality and premature disability attributable to high body mass index (BMI) (2). Cancer is a set of diseases caused by the interplay between genetic and environmental or behavioral factors, with high BMI being the third attributable behavioral risk factor behind smoking and infection globally and the second behind smoking in Western countries (3). Numerous large-scale epidemiological studies provide consistent and compelling evidence supporting a strong and potentially causal association between excess body weight and the risk of several types of cancer (4). Furthermore, obesity might also promote the progression of established cancer, impair the efficacy of therapy for cancer, and shorten the survival of patients with cancer (5).

Although it represents an urgent issue that needs to be properly addressed and a modifiable behavioral factor, high BMI rarely receives the attention it deserves in the oncology field. Great emphasis is given to the biological aspects of cancer during both medical training and decision-making for public health policy (6). Successfully addressing the challenge of cancer attributable to high BMI requires reliable and timely quantitative information on regional and global scales. Despite studies only assessed the global incidence of cancer attributable to high BMI based on data from GLOBOCAN in 2012, the currently available information is sparse (7, 8). The collaborative groups of the Global Burden of Disease (GBD) 2019 systematically quantified the burden of diseases attributable to high BMI by deaths and disability-adjusted life-years (DALYs), which provides an opportunity to update our information on the impact of high BMI on cancer. Several studies on non-communicable diseases (including cancer) and several individual cancer types have been published (2, 9–11). In this study, utilizing data obtained from GBD, we sought to comprehensively estimate the burden of cancer-related deaths and DALYs attributable to high BMI at the global, regional, and country levels in 2019. Additionally, we assessed the correlation between cancer burden and Socio-demographic Index (SDI). The long-term trends of the age-standardized mortality rate (ASMR) were also analyzed. The primary purpose of this study was to use these data and their variations to inform potential directions in primary prevention, screening, early diagnosis, and therapy of cancer attributable to high BMI.

## Materials and methods

### Data sources and search parameters

In this study, the research subjects were patients diagnosed with cancer attributable to high BMI. We obtained all data analyzed in this study from GBD 2019, which aims to analyze health trends over time, compare variability among countries, and help to establish disease control strategies globally (12). GBD 2019 quantifies health loss, including 369 diseases and injuries, and for 87 risk factors in 204 countries and territories around the world. The GBD relevant data sources are identified from censuses, household surveys, civil registration and vital statistics, disease registries, health service use, air pollution monitors, satellite imaging, disease notifications, and other sources. Detailed methodologies of GBD 2019 and the comparative risk assessment specifically for high BMI have been described in previous studies (12, 13).

We collected data from the Global Health Data Exchange (GHDx; http://ghdx.healthdata.org/) *via* the freely available GBD Results Tools repository. The search parameters were “neoplasm” and specific cancer types attributable to high BMI for cause; “high BMI” for risk; “deaths, DALYs” for measurements; “1990–2010” for years; and “number and rate” for metrics. Data were downloaded at the sex, SDI, GBD region, and country levels. We followed the Guidelines for Accurate and Transparent Health Estimates Reporting guidelines for cross-sectional studies (14).

### Definitions

High BMI was defined as adults (age 20+ years) with BMI ≥25 kg/m^2^ and using thresholds from the International Obesity Task Force standards for children (aged <20 years) (13). Detailed information about the data sources for data incorporating on high BMI and the process of data selection and data inputs has been published previously (13). The DALY is a summary measure that quantifies the overall burden of disease, which represents the sum of years of life lost due to premature death and years lived with disability. One DALY can be regarded as the loss of 1 year in full health. The modeling strategies for estimating cause-specific deaths and DALYs have been described in detail elsewhere (12). SDI is a composite indicator of a country's lag-distributed income per capita, average years of schooling, and the fertility rate in females under the age of 25 years (12). From the minimum level to the maximum level of development, SDI value can range from 0 to 1. The 195 countries and territories were categorized according to SDI into five groups: high SDI (>0.81), high-middle SDI (0.70–0.81), middle SDI (0.61–0.69), low-middle SDI (0.46–0.60), and low SDI (<0.46).

### Statistical analysis

Detailed methods for the incorporation of data on high BMI and the estimation of mortality and DALYs have been reported in previous studies by GBD collaborators (12, 15). We analyzed mortality and DALYs descriptively by gender, country, region, and year, and we calculated the change rates between 1990 and 2019. We plotted the temporal trends of these measures from 1990 to 2019. To compare the changing trends of mortality of cancer attributable to high BMI among different populations, we utilized Joinpoint software (version 4.9.0.0) to determine the average annual percentage change (AAPC) and the annual percentage change (APC) for each period, with a maximum of two joinpoints using a generalized linear regression model for the natural logarithm of the ASMR. We established the statistical significance of the variation trend by their 95% confidential intervals (CIs). We considered AAPCs or APCs with a 95% CI of >0 to represent a significant rising trend, while we deemed those with a 95% CI of <0 to represent a significant falling trend; otherwise, they represented a stable ASIR or ASMR (16, 17).

## Results

### Global trends of cancer attributable to high BMI

**Figure 1:**
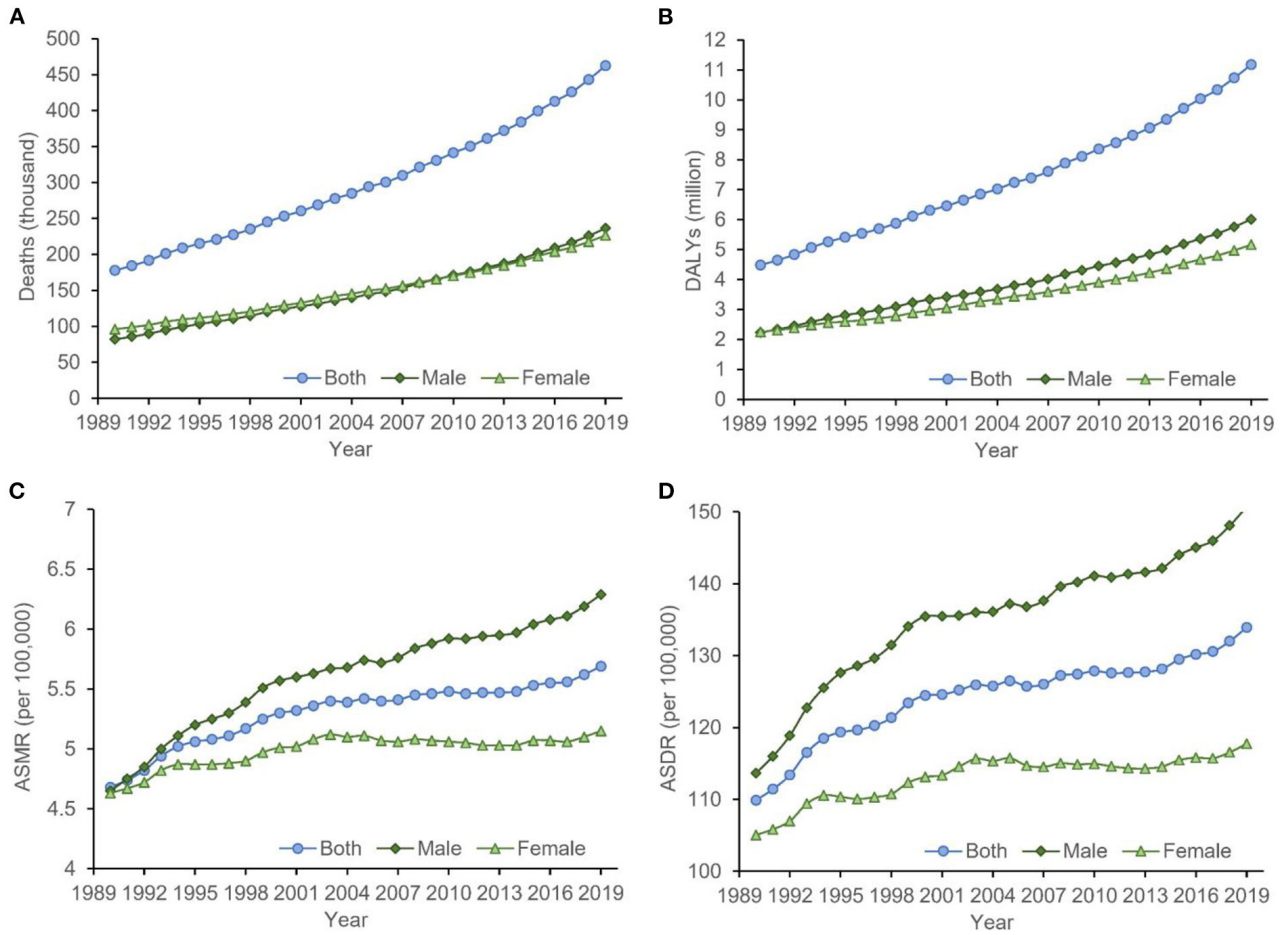
The temporal trends of the **(A)** deaths, **(B)** DALYs, **(C)** ASMR, and **(D)** ASDR for cancer attributable to high BMI from 1990 to 2019. The burden of cancer attributable to high BMI had been increasing significantly in both sexes, which was more obvious for men. ASDR, age-standardized DALYs rate; ASMR, age-standardized mortality rate; BMI, body mass index; DALYs, disability-adjusted life-years.

In 2019, there were more than one million cancer-related deaths worldwide, including 462.55 thousand (4.59%) deaths can be attributed to high BMI, corresponding to an ASMR of 5.69 per 100,000. DALYs resulting from cancer were estimated to be 251.39 million, of which 4.45% (11.18 million) was attributed to high BMI, with an age-standardized DALYs rate (ASDR) of 133.93 per 100,000. Between 1990 and 2019, the overall deaths, DALYs, ASMR, and ASDR increased by 160.17, 149.11, 21.58, and 21.88%, respectively. The global cancer-related deaths attributable to high BMI have increased from 81.80 thousand in 1990 to 236.27 thousand in 2019 for males and have increased from 95.98 thousand in 1990 to 22.63 thousand in 2019 for females. The global cancer-related DALYs attributable to high BMI have increased from 2.25 million in 1990 to 6.01 million in 2019 for males and have increased from 2.25 million in 1990 to 5.16 million in 2019 for females. From 1990 to 2019, the ASMRs of cancer attributable to high BMI increased by 35.27 and 11.23% for males and females, respectively, and the ASDRs of cancer attributable to high BMI increased by 32.61 and 12.13% for males and females, respectively (Table 1). Time trend analysis showed that the ASMR of cancer attributable to high BMI increased significantly, with an AAPC of 0.6 (0.5, 0.7). The majority of the increase was contributed by males, with the annual increasing rate being 1.0%, more than 3 times that for females (0.3%; Table 2, Figure 1).

**Table 1:** Global deaths and DALYs of cancer attributable to high body mass index in 2019 and percentage change from 1990 to 2019 by sex, SDI, and GBD region.

**Sex/SDI/GBD region**	**Death (thousand)**			**ASMR (per 100,000)**			**DALYs (thousand)**			**ASDR (per 100,000)**		
	**1990**	**2019**	**% change 1990–2019**	**1990**	**2019**	**% change 1990–2019**	**1990**	**2019**	**% change 1990–2019**	**1990**	**2019**	**% change 1990–2019**
Overall	177.79	462.55	160.17%	4.68	5.69	21.58%	4486.17	11175.31	149.11%	109.89	133.93	21.88%
Sex												
Male	81.80	236.27	188.83%	4.65	6.29	35.27%	2236.51	6011.22	168.78%	113.66	150.73	32.61%
Female	95.98	226.27	135.74%	4.63	5.15	11.23%	2249.66	5104.09	129.55%	105.02	117.76	12.13%
SDI												
Low SDI	3.26	12.95	297.63%	1.40	2.56	82.86%	92.47	361.36	290.77%	35.40	64.16	81.24%
Low-middle SDI	7.87	39.29	399.43%	1.36	2.92	114.71%	222.49	1051.07	372.41%	33.84	72.76	115.01%
Middle SDI	33.28	124.99	275.62%	3.26	5.03	54.29%	958.78	3342.67	248.64%	83.81	126.04	50.39%
High-middle SDI	62.90	140.76	123.77%	5.96	6.89	15.60%	1614.28	3366.03	108.52%	145.59	164.54	13.02%
High SDI	70.39	144.30	105.00%	5.96	7.53	11.72%	1595.73	3047.58	90.98%	156.71	174.68	11.47%
GBD region												
Central Asia	3.36	5.85	73.96%	7.18	8.07	12.40%	90.94	162.17	78.33%	182.83	198.99	8.84%
Central Europe	13.13	23.71	80.63%	8.91	10.96	23.01%	320.59	523.60	63.32%	212.81	256.17	20.37%
Eastern Europe	19.87	31.39	57.99%	6.96	9.01	29.45%	513.35	762.90	48.61%	176.40	224.51	27.27%
Australasia	1.75	4.25	143.62%	7.49	8.44	12.68%	40.06	88.83	121.73%	173.88	190.25	9.41%
High-income Asia Pacific	7.03	16.06	128.42%	3.52	3.49	−0.85%	174.85	307.05	75.61%	84.49	79.08	−6.40%
High-income North America	26.85	60.84	126.61%	7.66	9.57	24.93%	618.79	1361.72	120.06%	285.48	227.01	22.44%
Southern Latin America	3.53	7.88	123.18%	7.75	9.37	20.90%	83.52	170.86	104.57%	179.59	208.44	16.71%
Western Europe	41.01	7.14	74.23%	7.04	7.56	7.39%	88.50	1378.25	55.73%	158.46	164.83	4.02%
Andean Latin America	8.92	3.51	293.35%	4.39	6.33	44.19%	24.46	88.65	254.23%	111.42	151.58	36.04%
Caribbean	1.28	3.42	167.93%	4.97	6.58	32.39%	32.53	84.21	158.86%	122.32	161.57	32.09%
Central Latin America	3.25	12.13	273.11%	4.00	5.81	29.50%	86.59	304.67	251.83%	96.96	125.77	29.71%
Tropical Latin America	4.12	15.87	285.70%	4.66	6.59	41.42%	109.74	388.53	254.03%	112.97	156.41	38.45%
North Africa and Middle East	6.74	26.10	287.49%	3.98	6.25	57.04%	188.74	697.36	269.48%	99.87	150.07	50.27%
South Asia	4.41	30.60	594.47%	0.82	2.21	169.51%	123.20	814.82	561.39%	19.92	54.90	175.60%
East Asia	31.04	103.77	234.36%	3.49	4.98	42.69%	914.08	2757.87	201.71%	93.23	127.85	37.13%
Oceania	0.14	0.44	216.62%	4.45	5.91	32.81%	4.36	13.68	213.85%	123.22	161.21	30.83%
Southeast Asia	4.41	27.13	514.83%	1.66	4.35	162.05%	136.42	784.51	475.08%	46.06	115.76	151.32%
Central Sub-Saharan Africa	0.56	1.44	158.94%	2.49	2.81	12.85%	15.73	405.47	157.70%	62.06	69.09	11.33%
Eastern Sub-Saharan Africa	1.32	5.77	337.32%	1.78	3.64	104.49%	38.01	162.34	327.13%	45.52	90.55	98.92%
Southern Sub-Saharan Africa	1.87	5.24	175.06%	6.99	9.63	37.77%	51.31	131.64	156.58%	174.05	222.87	28.05%
Western Sub-Saharan Africa	1.26	5.79	359.91%	1.47	3.27	122.45%	33.88	153.10	351.89%	36.20	77.14	113.09%

ASDR, age-standardized DALY rate; ASMR, age-standardized mortality rate; DALYs, disability-adjusted life-years; SDI, sociodemographic index.

**Table 2:** Time trend analysis of ASMR for cancer attributable to high body mass index from 1990 to 2019 by sex, SDI, and GBD region.

**Sex/SDI/GBD region**	**Trend 1**		**Trend 2**		**Trend 3**		**AAPC**
	**Period**	**APC**	**Period**	**APC**	**Period**	**APC**	
Overall	1990–1994	1.7 (1.2, 2.3)	1994–2001	0.9 (0.6, 1.2)	2001–2019	0.3 (0.2, 0.3)	0.6 (0.5, 0.7)
Sex
Male	1990–1994	2.4 (1.9, 2.9)	1994–2000	1.4 (1.1, 1.8)	2000–2019	0.6 (0.5, 0.6)	1.0 (0.9, 1.1)
Female	1990–2004	0.7 (0.6, 0.8)	2003–2014	−0.2 (−0.3, 0)	2014–2019	0.4 (0, 0.8)	0.3 (0.2, 0.4)
SDI
Low SDI	1990–1997	1.0 (0.8, 1.2)	1997–2011	2.4 (2.3, 2.5)	2011–2019	2.7 (2.5, 2.9)	2.1(2.0, 2.2)
Low-middle SDI	1990–2007	2.8 (2.7, 2.8)	2007–2010	2.0 (−0.4, 4.5)	2010–2019	3.0 (2.8, 3.2)	2.8 (2.5, 3.0)
Middle SDI	1990–2001	2.2 (2.1, 2.3)	2001–2016	0.9 (0.9, 1.0)	2016–2019	1.9 (1.2, 2.5)	1.5 (1.4, 1.6)
High-middle SDI	1990–1994	2.4 (1.8, 3.1)	1994–2004	0.3 (0.2, 0.5)	2004–2019	0 (−0.1, 0.1)	0.5 (0.4, 0.6)
High SDI	1990–2001	1.1 (1.0, 1.1)	2001–2017	−0.2 (−0.2, −0.1)	2017–2019	0.9 (−0.2, 1.9)	0.4 (0.3, 0.4)
Region
Central Asia	1990–1994	1.3 (0.5, 2.2)	1994–2000	−2.0 (−2.6, −1.5)	2000–2019	1.1 (1.0, 1.2)	0.5 (0.3, 0.7)
Central Europe	1990–2001	0.8 (0.7, 0.9)	2001–2008	1.3 (1.0, 1.6)	2008–2019	0.1 (0, 0.3)	0.7 (0.6, 0.8)
Eastern Europe	1990–1994	6.0 (4.0, 8.1)	1994–1997	−3.4 (−9.1, 2.5)	1997–2019	0.8 (0.6, 0.9)	1.0 (0.4, 1.7)
Australasia	1990–1996	1.2 (0.9, 1.5)	1996–2017	0.1 (0.1, 0.2)	2017–2019	0.8 (−1.0, 2.7)	0.4 (0.3, 0.6)
High-income Asia Pacific	1990–2000	1.7 (1.6–1.8)	2000–2017	−1.2 (−1.3, −1.2)	2017–2019	1.3 (−0.3, 3.1)	−0.1 (−0.2, 0.1)
High-income North America	1990–1995	2.0 (1.7, 2.2)	1995–2002	1.1 (0.9, 1.3)	2002–2019	0.3 (0.2, 0.3)	0.8 (0.7, 0.8)
Southern Latin America	1990–2002	1.7 (1.5, 1.8)	2002–2014	−0.5 (−0.6, −0.4)	2014–2019	1.1 (0.6, 1.6)	0.7 (0.6, 0.8)
Western Europe	1990–2002	1.0 (0.9, 1.0)	2002–2016	−0.4 (−0.5, −0.4)	2016–2019	0.2 (−0.4, 0.7)	0.2 (0.1, 0.3)
Andean Latin America	1990–2001	0.9 (0.6, 1.2)	2001–2011	2.0 (1.5, 2.4)	2011–2019	0.6 (0.1, 1.1)	1.2 (1.0, 1.4)
Caribbean	1990–2000	−0.7 (−0.9, −0.4)	2000–2005	2.4 (1.4, 3.5)	2005–2019	1.7 (1.6, 1.9)	1.0 (0.8, 1.2)
Central Latin America	1990–1995	0.9 (0,4 1.4)	1995–2011	0.7 (0.6, 0.8)	2011–2019	1.4 (1.1, 1.7)	0.9 (0.8, 1.0)
Tropical Latin America	1990–2004	2.0 (1.9, 2.1)	2004–2013	1.0 (0.8, 1.2)	2013–2019	0 (−0.3, 0.3)	1.3 (1.2, 1.4)
North Africa and Middle East	1990–2002	0.8 (0.6, 0.9)	2002–2011	2.6 (2.4, 2.9)	2011–2019	1.5 (1.2, 1.8)	1.5 (1.4, 1.7)
South Asia	1990–2005	4.6 (4.3, 4.8)	2005–2011	1.3 (0.1, 2.6)	2011–2019	3.6 (3.0, 4.2)	3.6 (3.3, 3.9)
East Asia	1990–2001	2.3 (2.1, 2.4)	2001–2016	0 (0, 0.1)	2016–2019	3.3 (2.4, 4.3)	1.2 (1.1, 1.3)
Oceania	1990–2004	1.6 (1.5, 1.7)	2004–2013	−0.2 (−0.4, 0)	2013–2019	1.11 (0.9, 1,4)	0.9 (0.9, 1.0)
Southeast Asia	1990–1996	4.0 (3.5, 4.5)	1996–2014	3.4 (3.3, 3.5)	2014–2019	2.7 (2.1, 3.3)	3.4 (3.3, 3.6)
Central Sub-Saharan Africa	1990–1996	−0.1 (−0.6, 0.4)	1996–2008	−2.0 (−2.2, −1.9)	2008–2019	3.3 (3.1, 3.5)	0.4 (0.2, 0.5)
Eastern Sub-Saharan Africa	1990–1995	0.4 (0.1, 0.7)	1995–2001	2.2 (1.9, 2.6)	2001–2019	3.2 (3.2, 3.3)	2.5 (2.4, 2.6)
Southern Sub-Saharan Africa	1990–1995	3.6 (2.3, 5.0)	1995–1998	8.3 (2.2, 14.8)	1998–2019	−0.2 (−0.4, −0.1)	1.3 (0.7, 1.9)
Western Sub-Saharan Africa	1990–2007	2.8 (2.7, 2.9)	2007–2013	3.2 (2.8, 3.6)	2013–2019	2.2 (1.9, 2.5)	2.8 (2.7, 2.9)

AAPC, average annual percentage change; APC, annual percentage change; ASMR, age-standardized mortality rate; SDI, sociodemographic index.

### Geographical variations in cancer attributable to high BMI

Across 21 GBD regions, the heaviest burden of cancer attributable to high BMI occurred in East Asia (103.77 thousand deaths and 2.76 million DALYs), followed by Western Europe (71.44 thousand deaths and 1.39 million DALYs) and high-income North America (60.84 thousand deaths and 1.36 million DALYs). There were significant variations in the ASMR and ASDR for cancer attributable to high BMI. The highest age-standardized rates of cancer-related deaths and DALYs attributable to high BMI were observed in Central Europe (10.96 deaths and 256.17 DALYs per 100,000). The lowest age-standardized rates of cancer-related deaths and DALYs attributable to high BMI were observed in South Asia (2.21 deaths and 54.90 DALYs per 100,000). The number of deaths and DALYs caused by cancer attributable to high BMI increased in all GBD regions, with the top three increased regions being South Asia (594.47 for deaths and 561.39% for DALYs), Southeast Asia (514.83 for deaths and 475.08% for DALYs), and Western Sub-Saharan Africa (359.91 for deaths and 351.89% for DALYs; Table 1). The AAPC analysis showed annual increases of 3.6, 3.4, and 2.8% in the ASMR in the three GBD regions. Nearly all other GBD regions also showed an upward trend in the ASMR of cancer attributable to high BMI, except for high-income Asia Pacific region, which showed a stable trend between 1990 and 2019, mainly contributed by a significantly annual 1.2% decrease from 2000 to 2017 (Table 2).

### Country-level burden of cancer attributable to high BMI

China and the United States of America were the two leading countries in terms of cancer-related deaths attributable to high BMI, with 100.44 thousand and 55.10 thousand deaths in 2019, respectively. Together, they accounted for 32.63% of the total estimated deaths, followed by India (23.55 thousand), the Russian Federation (21.88 thousand), Germany (16.22 thousand), and Brazil (15.57 thousand). The DALYs followed the patterns of deaths, with China (2.67 million), the United States of America (1.24 million), and India (0.63 million) as the three leading countries. Together, they accounted for 40.58% of the total DALYs caused by cancer attributable to high BMI. In terms of ASMR and ASDR, Mongolia ranked first, with 25.93 deaths and 611.78 DALYs per 100,000, whereas Bangladesh ranked last, with 1.16 deaths and 30.19 DALYs per 100,000. All 204 countries and territories showed an increased number of deaths and DALYs between 1990 and 2019, and the three countries with the highest percentage changes were the United Arab Emirates, Equatorial Guinea, and Djibouti, all of which increased by more than 1,000% (Supplementary Table S1). The time trend analysis showed an upward trend of ASMR in 170 countries and territories, a significant downward trend only in 8 countries and territories and a stable trend in the other 26 countries and territories. The ASMR of cancer attributable to high BMI in 119 countries and territories increased by more than 1% annually, of which 31 countries and territories increased by more than 3% annually. The three countries with the highest increasing rates in the ASMR are Equatorial Guinea, Cabo Verde, and Vietnam, with AAPCs of 6.6 (6.2, 6.9), 5.4 (4.8, 6.1), and 4.8 (4.7, 5.0), respectively (Supplementary Table S1).

### Association between SDI and burden of cancer attributable to high BMI

**Figure 2:**
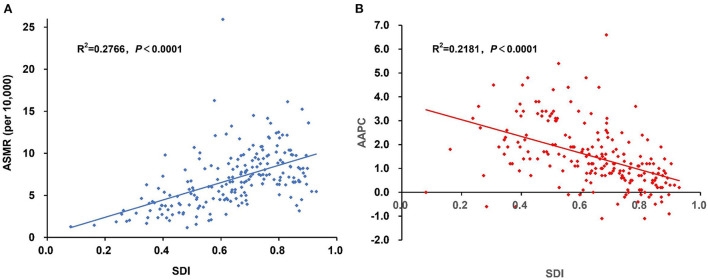
The association between SDI and the **(A)** ASMR and **(B)** AAPC of cancer attributable to high BMI in 2019. Pearson correlation analysis reflected a significantly positive correlation between the ASMR and SDI level, whereas a significantly negative correlation was found between the AAPC and SDI level. AAPC, average annual percentage change; ASMR, age-standardized mortality rate; BMI, body mass index; SDI, Socio-demographic Index.

The SDI-based regional analysis showed that the burden of cancer attributable to high BMI increased along with the increasing of SDI level. The number of deaths and DALYs in the high-SDI region was ~10 times that in the low-SDI region, whereas the age-standardized rates in the high-SDI region were ~four times for deaths and three times for DALYs that in the low-SDI region. In contrast, the percentage change and AAPC decreased along with the increasing of SDI level, with the highest increase occurring in the low-middle SDI region, which increased by 2.8% annually for the ASMR. In addition, the ASMR of cancer attributable to high BMI has decreased in the high SDI region since 2001 and has been stable in the high-middle SDI region since 2004 (Tables 1, 2). This pattern was also present at the country level (Supplementary Tables S1, S2). The Pearson correlation analysis reflected a significantly positive correlation between the ASMR and SDI level (*R*^2^ = 0.2766, *p* < 0.0001), whereas a significantly negative correlation was found between the AAPC and SDI level (*R*^2^ = 0.2181, *p* < 0.0001; Figure 2).

### Impact of high BMI on each cancer

**Figure 3:**
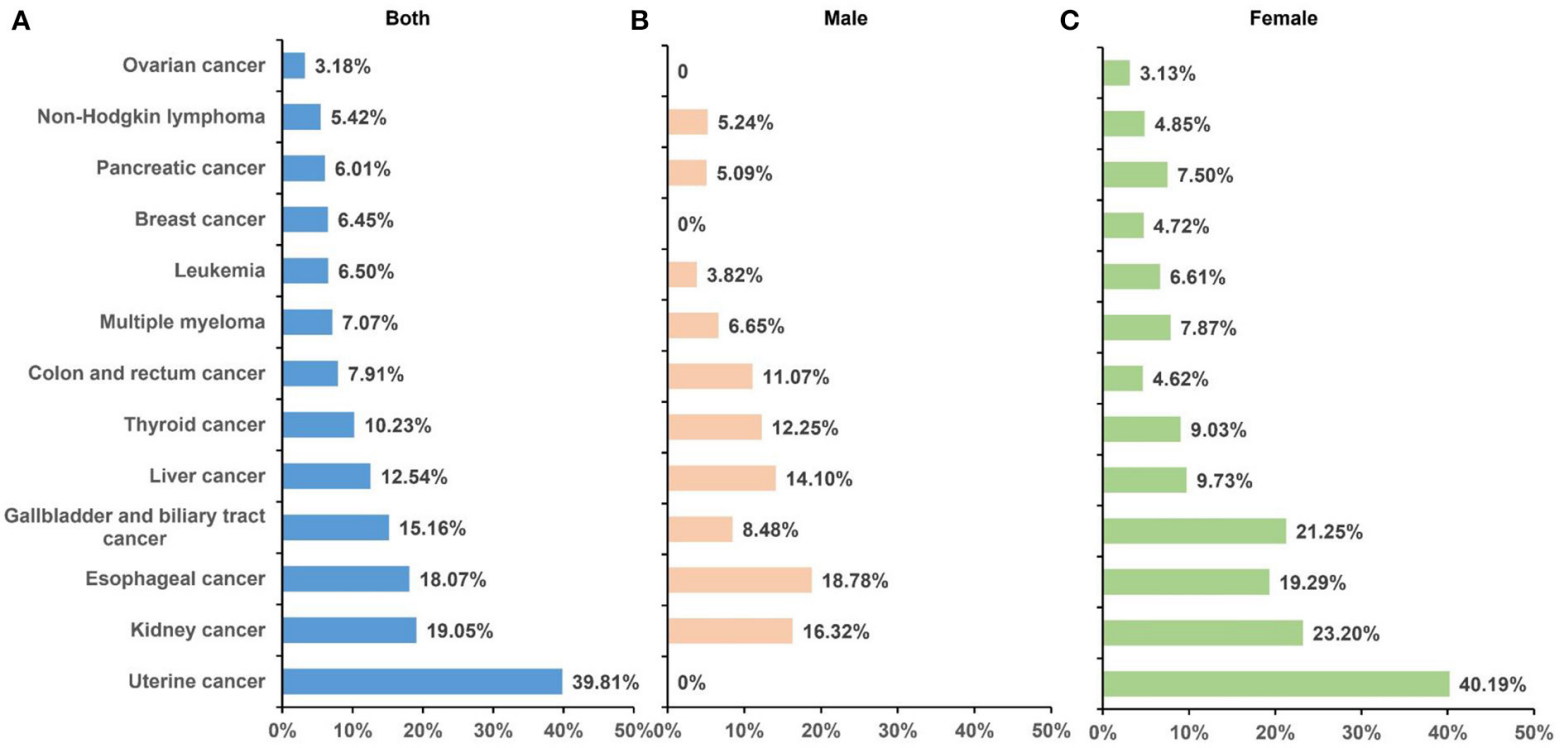
Fraction of DALYs of cancer attributable to high BMI by sex in 2019. A total of 13 cancer types were found to be affected by high BMI, and the contribution of high BMI varies among sexes and cancer types. **(A)** Both sexes; **(B)** male; **(C)** and female. BMI, body mass index; DALYs, disability-adjusted life-years.

In GBD 2019, 13 cancer types were found to be affected by high BMI, including esophageal cancer, breast cancer, gallbladder and biliary tract cancer, liver cancer, uterine cancer, pancreatic cancer, multiple myeloma, colon and rectum cancer, thyroid cancer, kidney cancer, ovarian cancer, non-Hodgkin lymphoma, and leukemia. In 2019, esophageal cancer was the leading cause of high BMI-related DALYs (26.27 per 100,000), followed by colorectal (24.21 per 100,000), liver (19.24 per 100,000), breast (11.22 per 100,000), and uterine cancer (11.22 per 100,000); they together accounted for 69.18% of all cancer-related DALYs attributable to high BMI. Similar patterns were observed for deaths. The fraction of DALYs attributable to high BMI was highest for uterine cancer (39.81%), followed by kidney cancer (19.05%) and esophageal cancer (18.07%). Between 1990 and 2019, the deaths and DALYs increased for all cancer types, and the greatest percentage changes in deaths and DALYs were for pancreatic cancer (229.32%) and breast cancer (231.79%; Table 3, Figure 3). The ASMR increased for cancer types except for gallbladder and biliary tract cancer and uterine cancer, which showed a decrease of −5.71 and −2.17%, respectively. Time trend analysis showed that the largest increase in ASMR was seen in thyroid cancer (AAPC 1.7%), followed by pancreatic cancer (AAPC 1.4%) and kidney cancer (AAPC 1.1%). Although AAPC showed a stable trend for ASMR of gallbladder and biliary tract cancer and uterine cancer, a significantly decreasing trend was found for gallbladder and biliary tract cancer (APC −0.8%) since 2007 and for uterine cancer (APC −0.2%) since 2003 (Table 4).

**Table 3:** Global deaths and DALYs of cancer attributable to high body mass index in 2019 and percentage change from 1990 to 2019 by cancer type.

**Cancer type**	**Death (thousand)**			**ASMR (per 100,000)**			**DALYs (thousand)**			**ASDR (per 100,000)**		
	**1990**	**2019**	**% change 1990–2019**	**1990**	**2019**	**% change 1990–2019**	**1990**	**2019**	**% change 1990–2019**	**1990**	**2019**	**% change 1990–2019**
Esophageal cancer	35.29	89.90	154.81%	0.90	1.09	21.11%	917.93	2202.31	139.92%	22.26	26.27	18.01%
Breast cancer	15.21	45.20	197.19%	0.42	0.55	30.95%	288.79	958.19	231.79%	7.86	11.22	42.75%
Gallbladder and biliary tract cancer	12.71	26.13	105.58%	0.35	0.33	−5.71%	284.06	567.75	99.87%	7.17	6.86	−4.32%
Liver cancer	23.18	60.80	162.34%	0.57	0.74	29.82%	678.62	1613.28	137.73%	15.91	19.24	20.93%
Uterine cancer	17.19	36.49	112.27%	0.46	0.45	−2.17%	444.33	935.96	110.64%	10.93	11.22	2.65%
Pancreatic cancer	9.69	31.92	229.32%	0.26	0.40	53.85%	224.25	709.45	216.36%	5.60	8.54	52.50%
Multiple myeloma	2.78	8.02	188.36%	0.07	0.10	42.86%	65.47	179.77	174.60%	1.64	2.17	32.32%
Colon and rectum cancer	31.90	85.88	169.26%	0.86	1.07	24.42%	769.65	2021.54	162.66%	19.14	24.41	27.53%
Thyroid cancer	1.68	4.66	177.61%	0.04	0.06	50.00%	45.99	127.59	177.45%	1.11	1.54	38.74%
Kidney cancer	11.20	31.70	183.16%	0.29	0.39	34.48%	285.055	751.89	163.77%	6.97	9.05	29.84%
Ovarian cancer	2.63	6.31	139.88%	0.07	0.08	14.29%	70.87	167.92	136.94%	1.71	2.00	16.96%
Non-Hodgkin lymphoma	4.99	13.79	176.17%	0.13	0.17	30.77%	137.25	355.58	159.07%	3.26	4.30	31.90%
Leukemia	9.35	21.73	132.50%	0.24	0.27	12.50%	273.90	584.09	113.25%	6.33	7.10	12.16%

ASDR, age-standardized DALY rate; ASMR, age-standardized mortality rate; DALYs, disability-adjusted life-years.

**Table 4:** Time trend analysis of ASMR for cancer attributable to high body mass index from 1990 to 2019 by cancer type.

**Cancer type**	**Trend 1**		**Trend 2**		**Trend 3**		**AAPC**
	**Period**	**APC**	**Period**	**APC**	**Period**	**APC**	
Esophageal cancer	1990–2005	1.7 (1.6, 1.8)	2005–2015	−1.0 (−1.3, −0.8)	2015–2019	1.1 (0.1, 2.1)	0.7 (0.5, 0.8)
Breast cancer	1990–2001	1.0 (0.8, 1.1)	2001–2004	1.4 (−0.8, 3.7)	2004–2019	0.7 (0.7, 0.8)	0.9 (0.7, 1.1)
Gallbladder and biliary tract cancer	1990–2007	−0.2 (−0.3, −0.1)	2007–2016	−0.8 (−1.1, −0.4)	2016–2019	1.1 (−0.6, 2.9)	−0.2 (−0.4, 0)
Liver cancer	1990–1999	3.1 (2.8, 3.3)	1999–2004	−4.2 (−4.9, −3.5)	2004–2019	1.2 (1.1, 1.3)	0.8 (0.7, 1.0)
Uterine cancer	1990–2000	0 (−0.3, 0.2)	2000–2003	0.7 (−2.5, 4.0)	2003–2019	−0.2 (−0.4, −0.1)	−0.1 (−0.4, 0.3)
Pancreatic cancer	1990–2012	1.5 (1.4, 1.6)	2012–2017	0.7 (−0.6, 2.0)	2017–2019	2.4 (−1.8, 6.7)	1.4 (1.1, 1.8)
Multiple myeloma	1990–1999	2.2 (1.3, 3.2)	1999–2004	−0.1 (−3.3, 3.1)	2004–2019	0.9 (0.5, 1.4)	1.1 (0.5, 1.8)
Colon and rectum cancer	1990–2004	1.0 (0.9, 1.2)	2004–2016	0.2 (0, 0.4)	2016–2019	1.2 (−0.2, 2.6)	0.7 (0.6, 0.9)
Thyroid cancer	1990–1992	13.8 (3.0, 25.7)	1992–2009	0.1 (−0.3, 0.4)	2009–2019	2.3 (1.5, 3.1)	1.7 (1.0, 2.5)
Kidney cancer	1990–1993	3.5 (2.1, 4.9)	1993–2008	1.1 (1.0, 1.2)	2008–2019	0.3 (0.2, 0.5)	1.1 (0.9, 1.2)
Ovarian cancer	1990–2010	0 (−0.2, 0.2)	2010–2016	0.8 (−0.7, 2.4)	2016–2019	3.7 (0.1, 7.3)	0.5 (0.1, 1.0)
Non-Hodgkin lymphoma	1990–1998	2.5 (2.0, 3.1)	1998–2011	0.1 (−0.3, 0.4)	2011–2019	0.9 (0.4, 1.5)	1.0 (0.7, 1.2)
Leukemia	1990–1992	1.7 (−1.8, 5.3)	1992–2003	0.6 (0.4, 0.9)	2003–2019	0.2 (0, 0.3)	0.4 (0.2, 0.7)

AAPC, average annual percentage change; APC, annual percentage change; ASMR, age-standardized.

## Discussion

Overall, the global mortality of cancer attributable to high BMI was 5.69 per 100,000 people per year, with an estimated 462.55 thousand deaths, and the corresponding ASDR was 133.93 per 100,000 people per year, with an estimated 11.18 million DALYs in 2019. The estimated number of high BMI-related cancer cases accounted for 4.59 and 4.45% of all cancer-cause deaths and DALYs, respectively. From 1990 to 2019, both deaths and DALYs of cancer attributable to high BMI have more than doubled worldwide. After age-standardizing the rates of mortality and DALYs, the percentage changes were much lower than those of numbers; however, an annual 0.6% increase was observed for ASMR, and the rate of increase slowed since 2000. The marked increase in the number of global cancer-related deaths and DALYs attributable to high BMI can be partially explained by the increase in the prevalence of high BMI and the growth and aging of the population globally. During recent decades, despite a better understanding of obesity pathogenesis and its health challenge, high BMI has not shown a declining pattern but rather an upward trend, leading to its continuous pandemic worldwide, with 40% of adults and 18% of children being determined to be obese or overweight in 2016 (18–20). Notably, the prevalence of obesity has quadrupled for men and more than doubled for women, resulting in 671 million obese adults worldwide (20). The prevalence of high BMI was higher among older populations, along with aging and increasing life expectancy, leading to a substantial increase in the numbers of deaths and DALYs caused by high BMI-related cancer (3). However, the disease burden of cancer attributable to high BMI varies widely according to sex, geographical region, country, and SDI level, which is related to the differences in genetic predispositions, prevalence of high BMI, case fatality of cancer, and exposure to other risk factors.

In general, the burden of cancer attributable to high BMI was heavier in regions with higher SDI levels, with nearly two-thirds of deaths occurring in high- and middle-SDI regions, whereas the rate of increase slowed or even showed a decreasing trend in these regions in the recent years. In contrast, in regions with lower SDI levels, although the baseline burden of cancer attributable to high BMI was relatively low, both the numbers and rates of deaths and DALYs showed a significantly increasing trend and may not stop increasing for a period of time. The differences in SDI may also contribute to the variations in high BMI-related cancer burden at the GBD region and country levels. This was parallel with the distribution and changing pattern of high BMI, for which national wealth is the most apparent systematic driver (21, 22). The increasing prevalence of high BMI began in the 1970s in high-income Western countries, in which the absolute increase was highest whereas the prevalence showed a possible plateau in the recent years (20). The beginning time of increasing prevalence of high BMI was later in low-income and middle-income countries, which were historically low-risk regions, whereas the relative increase was high, and the prevalence has increased rapidly in these countries (20). This shift may be mainly attributed to the introduction of the “Western lifestyle,” consisting of high intake of energy-dense and nutrient-poor foods along with reduced physical activity levels (23). However, several exceptions also exist. For example, obesity prevalence is quite low in high-income Asian Pacific countries, leading to their small increasing or slightly decreasing ASMR of high BMI-related cancer, which is likely a result of adherence to traditional habits and an active transportation system (24).

In GBD 2019, 13 cancer types were found to be affected by high BMI, the majority of which were also found to be associated with excess body weight in other studies (4, 25). However, inconsistency also exists. For example, cancer of gastric cardia was listed as a strong evidence-supported obesity-related cancer by the International Agency for Research on Cancer (IARC) group but not in GBD 2019 (25). The reasons for this inconsistency may be different adiposity indices (BMI, waist circumference, and waist-to-hip ratio) and cancer classification systems utilized among different study groups. The extent of the effects of high BMI on mortality varied across cancer sites, reflecting the variation in relative risks associated with high BMI. High BMI accounted for ~39.81% of deaths caused by endometrial cancer, compared with only 3.18% of ovarian cancer, which was consistent with the proportions of incidence of cancer caused by high BMI estimated by Sung et al., and was also consistent with the relative risk found by the IARC group, which was 7.1 for cancer of corpus uteri, 4.8 for esophageal adenocarcinoma, and only 1.1 for ovarian cancer (7, 25). Various mechanisms through which fatness affects cancer risk have been proposed, with hormonal alteration and chronic inflammation being the most studied hypotheses (26, 27). Metabolic hormonal alterations and chronic inflammation have been linked to multiple types of cancer, whereas sex-steroid hormonal alterations may affect hormone-sensitive cancers. Although the causal relationship between high BMI and poor prognosis has not been robustly established, numerous studies have reported that high BMI portends unfavorable outcomes for several cancers (28, 29). In addition to the mechanisms participating in carcinogenesis, which also promote the progression of cancer, impairing the efficacy of cancer therapy and obesity-related comorbidities also contributes to the poor prognosis of obese patients with cancer (5, 30).

In GBD 2019, the burden of cancer attributable to high BMI was measured by mortality and DALYs, which reflect variations not only in incidence but also in case fatality (31). Case fatality can be affected by several factors, including early diagnosis, accessibility to treatment, tumor biological behavior, and efficacy of cancer management. Therefore, the rapidly increasing pace of mortality of high BMI-related cancer in less developed countries is likely due to not only the rapidly increasing prevalence of high BMI but also the fractured health infrastructures, the detection of cancers at a later stage, and poor access to and availability of treatment (32). Case fatality may also explain the variations in incidence and mortality between sexes and across cancer types. In a 2012 report, the number of new cases of high BMI-related cancer in women was two times that in men, whereas the number of deaths was equal between males and females in GBD 2019 (7). This was because in males, the predominant cancer types were esophageal, colorectal, and liver cancer, which have high mortality-to-incidence ratios, whereas in females, the common cancer types, such as breast and uterine cancer, have relatively low mortality-to-incidence ratios (33).

As a result, mainly of behavioral changes, high BMI also presented as a modifiable risk factor for cancer. Therefore, compared with genetic predisposition and aging, decreasing the burden of cancer by policies and actions to prevent and control excess body weight may be easier, which is one of the targets of the World Health Organization in an effort to address the growing global burden of NCD (34). Although achieving this goal appears to be unlikely, evidence-based and cost-effective strategies consisting of a healthy diet and physical activity have been attempted in some countries, and their feasibility has been proven (35, 36). Compelling evidence indicating that exercise and weight loss are associated with lower disease risk and better survival for several cancer types was also found in observational studies (37, 38). In addition, long-term follow-up bariatric or metabolic surgery for morbid obesity or obesity complicated by T2DM provided indirect evidence, which showed that weight loss in the population with obesity reduces cancer risk (39–41). However, efforts to intervene effectively with weight-reduction strategies in the cancer population have not yet entered routine clinical practice.

Our results provide a comprehensive estimation of cancer attributable to high BMI, which may be a reference for establishing interventions to address this challenge and monitoring their effectiveness over time. However, this study also has several limitations. First, although GBD 2019 used many strategies to improve the data quality and comparability, bias is inevitable, which may affect the integrity and accuracy of the data that we analyzed. Second, BMI, despite being a popular metric, the percentage of body fat and the patterns of obesity varied obviously in individuals with the same BMI value, which can be attributed to age, sex, and ethnicity. Therefore, cutoffs utilized for BMI category may misestimate the effect of high BMI on cancer in specific populations. Third, data about specific subtypes of cancer and some cancer types with compelling evidence supporting their association with high BMI are not available in GBD 2019. Fourth, carcinogenesis is a multifactorial process, and other factors may interact with high BMI to influence the risk of cancer development. For example, the risk of female hormone-driven cancers related to high BMI is largely attenuated or even eliminated among hormone replacement therapy (HRT) users, whereas the risk of pancreatic and thyroid cancers related to high BMI is attenuated by smoking (7). Therefore, the burden of these cancer types might be underestimated in regions with high HRT and smoking prevalence. Finally, in GBD 2019, information about high BMI prevalence and cancer mortality was obtained for the same year, not allowing for a lag between the exposure and cancer development and mortality.

## Conclusion

Although the effects of high BMI on cancer risk are only found in specific cancer types and are modest for most cancer sites, high BMI is highly prevalent in high-income countries and has been increasing continuously across the majority of population groups, especially in less developed countries with ineffective cancer management, leading to a substantial burden of high BMI-related cancer. Although compelling evidence indicates that exercise and weight loss are associated with lower disease risk and better survival for many cancer types, efforts to intervene effectively with weight-reduction strategies in the cancer population have not yet entered routine clinical practice. Therefore, concerted action by governments, stakeholders, civil societies, health-care providers, and individuals should be suggested to increase the awareness of the harmful effects of high BMI, promote a healthy diet and physical activity, and improve health care for obese populations, aiming to decrease the burden of disease attributed to high BMI, including cancer.

## Data availability statement

The original contributions presented in the study are included in the article/Supplementary material, further inquiries can be directed to the corresponding author.

## Ethics statement

This study was approved by the Academic Committee of the Third Hospital of Mianyang (20190307). Because GBD 2019 uses de-identified, aggregated data, informed consent was waived for this study.

## Author contributions

All authors listed have made a substantial, direct, and intellectual contribution to the work and approved it for publication.

## Funding

This study was supported by Scientific Research Projects of Health Commission of Mianyang City (202012).

## Conflict of interest

The authors declare that the research was conducted in the absence of any commercial or financial relationships that could be construed as a potential conflict of interest.

## Publisher's note

All claims expressed in this article are solely those of the authors and do not necessarily represent those of their affiliated organizations, or those of the publisher, the editors and the reviewers. Any product that may be evaluated in this article, or claim that may be made by its manufacturer, is not guaranteed or endorsed by the publisher.
