# Supplementary material for: The global burden and temporal trend of cancer attributable to high body mass index: Estimates from the Global Burden of Disease Study 2019
Source: Front Nutr. 2022 Jul 26;9:918330. doi: 10.3389/fnut.2022.918330 (PMC9360580; doi:10.3389/fnut.2022.918330)
Supplement: Supplementary file 1 [file Data_Sheet_1.docx]

Supplementary Material

**Supplementary Table 1.** Global deaths and DALYs of cancer attributable to high body mass index in 2019 and percentage change from 1990 to 2019 by country

| Country | Death | | | ASMR | | | DALYs | | | ASDR | | |
| --- | --- | --- | --- | --- | --- | --- | --- | --- | --- | --- | --- | --- |
|  | 1990 | 2019 | % change  1990-2019 | 1990 | 2019 | % change  1990-2019 | 1990 | 2019 | % change  1990-2019 | 1990 | 2019 | % change  1990-2019 |
| Armenia | 109.66 | 334.66 | 205.18% | 3.89 | 7.98 | 105.14% | 3002.89 | 7990.32 | 166.09% | 97.73 | 188.49 | 92.87% |
| Azerbaijan | 277.82 | 796.65 | 186.75% | 5.36 | 8.57 | 59.89% | 8011.35 | 22392.60 | 179.51% | 142.90 | 209.05 | 46.29% |
| Georgia | 362.97 | 429.25 | 18.26% | 5.73 | 7.25 | 26.53% | 9741.94 | 10710.30 | 9.94% | 147.24 | 190.50 | 29.38% |
| Kazakhstan | 1415.24 | 1,691.96 | 19.55% | 11.39 | 9.94 | -12.73% | 36914.06 | 44396.20 | 20.27% | 277.69 | 241.16 | -13.15% |
| Kyrgyzstan | 142.05 | 201.52 | 41.87% | 4.63 | 4.5 | -2.81% | 3825.12 | 5500.33 | 43.79% | 119.67 | 110.53 | -7.64% |
| Mongolia | 155.22 | 563.52 | 263.05% | 15.15 | 25.93 | 71.16% | 4182.75 | 16033.77 | 283.33% | 381.72 | 611.78 | 60.27% |
| Tajikistan | 79.35 | 175.28 | 120.89% | 2.80 | 3.6 | 28.57% | 2244.71 | 5207.48 | 131.99% | 74.74 | 88.55 | 18.48% |
| Turkmenistan | 198.40 | 307.94 | 55.21% | 10.35 | 7.76 | -25.02% | 5479.78 | 8888.27 | 62.20% | 265.80 | 202.60 | -23.78% |
| Uzbekistan | 621.48 | 1,348.22 | 116.94% | 5.42 | 6.59 | 21.59% | 17534.76 | 41049.54 | 134.10% | 143.78 | 163.97 | 14.04% |
| Albania | 88.56 | 231.65 | 161.57% | 4.44 | 5.34 | 20.27% | 2255.12 | 5275.63 | 133.94% | 102.94 | 124.79 | 21.23% |
| Bosnia and Herzegovina | 221.59 | 572.02 | 158.14% | 5.47 | 9.49 | 73.49% | 5868.20 | 13039.11 | 122.20% | 131.90 | 219.27 | 66.24% |
| Bulgaria | 985.07 | 1,387.15 | 40.82% | 7.74 | 9.8 | 26.61% | 25663.48 | 32931.79 | 28.32% | 200.55 | 252.39 | 25.85% |
| Croatia | 518.87 | 978.61 | 88.60% | 8.13 | 10.9 | 34.07% | 12673.11 | 20353.49 | 60.60% | 191.87 | 244.32 | 27.34% |
| Czechia | 1742.68 | 2,607.68 | 49.64% | 12.60 | 12.16 | -3.49% | 40434.86 | 54477.76 | 34.73% | 296.64 | 271.08 | -8.62% |
| Hungary | 1971.66 | 2,400.40 | 21.75% | 13.48 | 12.41 | -7.94% | 46383.54 | 52743.39 | 13.71% | 319.05 | 293.32 | -8.06% |
| Montenegro | 48.33 | 100.67 | 108.30% | 7.76 | 10.14 | 30.67% | 1225.63 | 2361.13 | 92.65% | 188.51 | 239.07 | 26.82% |
| North Macedonia | 124.67 | 333.21 | 167.27% | 6.68 | 10.4 | 55.69% | 3222.09 | 8032.48 | 149.29% | 162.31 | 243.11 | 49.78% |
| Poland | 4184.81 | 8,411.48 | 101.00% | 9.61 | 11.83 | 23.10% | 100001.44 | 181932.36 | 81.93% | 226.39 | 269.25 | 18.93% |
| Romania | 1462.86 | 3,193.03 | 118.27% | 5.11 | 8.76 | 71.43% | 38381.82 | 74613.44 | 94.40% | 131.38 | 220.42 | 67.77% |
| Serbia | 955.99 | 1,897.07 | 98.44% | 8.40 | 11.81 | 40.60% | 24548.05 | 42735.59 | 74.09% | 203.11 | 277.16 | 36.46% |
| Slovakia | 600.87 | 1,136.06 | 89.07% | 10.02 | 12.18 | 21.56% | 14818.03 | 25861.71 | 74.53% | 247.70 | 282.72 | 14.14% |
| Slovenia | 221.55 | 462.85 | 108.91% | 9.04 | 10.37 | 14.71% | 5118.65 | 9244.63 | 80.61% | 207.47 | 227.21 | 9.51% |
| Belarus | 672.24 | 1,200.23 | 78.54% | 5.06 | 7.47 | 47.63% | 17430.22 | 29363.78 | 68.46% | 129.19 | 186.68 | 44.50% |
| Estonia | 141.97 | 280.61 | 97.65% | 6.86 | 10.38 | 51.31% | 3492.81 | 5739.66 | 64.33% | 168.29 | 236.95 | 40.80% |
| Latvia | 242.84 | 404.32 | 66.50% | 6.69 | 10.07 | 50.52% | 5963.33 | 8668.31 | 45.36% | 164.35 | 238.73 | 45.26% |
| Lithuania | 297.28 | 572.37 | 92.54% | 6.54 | 9.85 | 50.61% | 7355.33 | 12107.20 | 64.60% | 161.19 | 228.95 | 42.04% |
| Republic of Moldova | 233.06 | 406.1 | 74.25% | 5.08 | 6.88 | 35.43% | 6403.73 | 10548.73 | 64.73% | 134.80 | 181.02 | 34.29% |
| Russian Federation | 12885.80 | 21,875.90 | 69.77% | 7.03 | 9.19 | 30.73% | 333252.01 | 527034.29 | 58.15% | 176.98 | 225.58 | 27.46% |
| Ukraine | 5394.04 | 6,649.18 | 23.27% | 7.33 | 8.76 | 19.51% | 139449.80 | 169439.52 | 21.51% | 188.42 | 231.58 | 22.91% |
| Australia | 1453.15 | 3,588.05 | 146.92% | 7.49 | 8.45 | 12.82% | 33370.28 | 74684.91 | 123.81% | 173.64 | 190.31 | 9.60% |
| New Zealand | 293.16 | 666.34 | 127.30% | 7.52 | 8.38 | 11.44% | 6691.48 | 14142.49 | 111.35% | 175.07 | 189.92 | 8.48% |
| Brunei Darussalam | 3.09 | 17.43 | 464.08% | 3.44 | 6.24 | 81.40% | 89.08 | 502.41 | 464.00% | 80.43 | 144.42 | 79.56% |
| Japan | 5969.55 | 11,740.97 | 96.68% | 3.53 | 3.15 | -10.76% | 144418.30 | 207154.10 | 43.44% | 83.90 | 69.40 | -17.28% |
| Republic of Korea | 996.02 | 4,002.97 | 301.90% | 3.36 | 4.49 | 33.63% | 28728.52 | 92303.70 | 221.30% | 84.69 | 102.65 | 21.21% |
| Singapore | 60.88 | 295.25 | 384.97% | 2.83 | 3.79 | 33.92% | 1616.72 | 7090.92 | 338.60% | 68.38 | 87.18 | 27.49% |
| Canada | 2149.81 | 5,725.59 | 166.33% | 6.66 | 8.14 | 22.22% | 49895.66 | 122108.55 | 144.73% | 156.99 | 185.72 | 18.30% |
| Greenland | 3.84 | 9.81 | 155.47% | 11.43 | 14.16 | 23.88% | 110.43 | 254.13 | 130.13% | 285.08 | 334.15 | 17.21% |
| United States of America | 24691.89 | 55,100.38 | 123.15% | 7.77 | 9.74 | 25.35% | 568769.56 | 1239333.13 | 117.90% | 188.54 | 232.05 | 23.08% |
| Argentina | 2264.89 | 4,963.04 | 119.13% | 7.08 | 9.14 | 29.10% | 53346.12 | 108382.16 | 103.17% | 163.15 | 205.49 | 25.95% |
| Chile | 943.23 | 2,337.20 | 147.79% | 9.80 | 9.68 | -1.22% | 22847.56 | 50610.59 | 121.51% | 223.23 | 210.07 | -5.90% |
| Uruguay | 320.94 | 575.71 | 79.38% | 8.20 | 10.46 | 27.56% | 7327.00 | 11859.98 | 61.87% | 190.64 | 236.79 | 24.21% |
| Andorra | 5.20 | 13.96 | 168.46% | 9.90 | 9.75 | -1.52% | 126.31 | 304.65 | 141.19% | 221.56 | 219.37 | -0.99% |
| Austria | 900.29 | 1,214.39 | 34.89% | 7.56 | 6.58 | -12.96% | 19299.98 | 23894.10 | 23.80% | 171.62 | 143.00 | -16.68% |
| Belgium | 1036.74 | 1,688.72 | 62.89% | 6.66 | 7.09 | 6.46% | 21494.06 | 32467.06 | 51.05% | 142.65 | 153.20 | 7.40% |
| Cyprus | 28.27 | 90.48 | 220.06% | 3.62 | 4.76 | 31.49% | 634.51 | 1931.10 | 204.35% | 77.27 | 100.39 | 29.92% |
| Denmark | 495.40 | 856.03 | 72.80% | 6.13 | 7.26 | 18.43% | 10598.42 | 17176.05 | 62.06% | 140.56 | 158.48 | 12.75% |
| Finland | 442.37 | 838.51 | 89.55% | 6.17 | 6.58 | 6.65% | 9609.95 | 16163.84 | 68.20% | 138.53 | 142.45 | 2.83% |
| France | 6070.71 | 10,247.48 | 68.80% | 7.36 | 7.17 | -2.58% | 131500.13 | 194568.54 | 47.96% | 169.54 | 157.57 | -7.06% |
| Germany | 9722.09 | 16,216.33 | 66.80% | 7.58 | 8.2 | 8.18% | 210316.95 | 313167.61 | 48.90% | 170.80 | 178.65 | 4.60% |
| Greece | 785.29 | 1,572.61 | 100.26% | 5.12 | 6.31 | 23.24% | 17120.95 | 29173.42 | 70.40% | 111.92 | 137.84 | 23.16% |
| Iceland | 19.81 | 37.64 | 90.01% | 6.92 | 6.64 | -4.05% | 433.92 | 790.83 | 82.25% | 158.22 | 150.60 | -4.82% |
| Ireland | 312.12 | 629.52 | 101.69% | 7.68 | 8.31 | 8.20% | 6798.54 | 13060.95 | 92.11% | 170.92 | 179.50 | 5.02% |
| Israel | 309.15 | 805.4 | 160.52% | 6.47 | 6.78 | 4.79% | 6468.39 | 15831.79 | 144.76% | 135.41 | 141.26 | 4.32% |
| Italy | 5731.10 | 9,483.12 | 65.47% | 6.39 | 6.3 | -1.41% | 126964.13 | 178405.84 | 40.52% | 145.69 | 137.75 | -5.45% |
| Luxembourg | 43.97 | 64.8 | 47.37% | 8.05 | 6.32 | -21.49% | 970.26 | 1319.07 | 35.95% | 180.90 | 136.34 | -24.63% |
| Malta | 20.63 | 48.09 | 133.11% | 4.88 | 5.07 | 3.89% | 459.47 | 979.22 | 113.12% | 107.45 | 112.46 | 4.66% |
| Monaco | 7.19 | 13.41 | 86.51% | 10.02 | 13.61 | 35.83% | 141.29 | 257.08 | 81.95% | 224.90 | 301.68 | 34.14% |
| Netherlands | 1305.70 | 2,884.86 | 120.94% | 6.50 | 8.21 | 26.31% | 27383.80 | 57613.25 | 110.39% | 141.20 | 176.02 | 24.66% |
| Norway | 339.96 | 541.48 | 59.28% | 4.94 | 5.47 | 10.73% | 7001.58 | 10764.86 | 53.75% | 111.73 | 118.47 | 6.03% |
| Portugal | 723.36 | 1,542.96 | 113.30% | 5.22 | 6.37 | 22.03% | 16493.55 | 30950.36 | 87.65% | 119.24 | 146.51 | 22.87% |
| San Marino | 2.31 | 5.19 | 124.68% | 6.92 | 7.66 | 10.69% | 48.16 | 97.56 | 102.57% | 147.05 | 164.06 | 11.57% |
| Spain | 3574.80 | 7,419.53 | 107.55% | 6.53 | 7.48 | 14.55% | 80134.97 | 144685.69 | 80.55% | 150.25 | 166.49 | 10.81% |
| Sweden | 944.40 | 1,449.72 | 53.51% | 6.19 | 6.62 | 6.95% | 19060.07 | 27352.70 | 43.51% | 137.25 | 141.38 | 3.01% |
| Switzerland | 559.74 | 988.99 | 76.69% | 5.33 | 5.45 | 2.25% | 11717.66 | 19038.60 | 62.48% | 119.11 | 116.64 | -2.07% |
| United Kingdom | 7590.35 | 12,726.00 | 67.66% | 8.33 | 9.81 | 17.77% | 159502.70 | 247053.71 | 54.89% | 185.01 | 209.72 | 13.36% |
| Bolivia (Plurinational State of) | 158.60 | 739.41 | 366.21% | 4.93 | 8.53 | 73.02% | 4442.43 | 18537.04 | 317.27% | 128.40 | 201.21 | 56.71% |
| Ecuador | 266.62 | 1,059.84 | 297.51% | 4.99 | 7.17 | 43.69% | 7511.65 | 26660.95 | 254.93% | 129.70 | 171.02 | 31.86% |
| Peru | 466.61 | 1,708.74 | 266.20% | 3.96 | 5.36 | 35.35% | 12506.79 | 41450.28 | 231.42% | 98.58 | 128.28 | 30.13% |
| Antigua and Barbuda | 2.19 | 6.41 | 192.69% | 4.21 | 6.39 | 51.78% | 51.92 | 160.00 | 208.17% | 105.04 | 150.04 | 42.84% |
| Bahamas | 13.41 | 34.61 | 158.09% | 8.81 | 8.84 | 0.34% | 371.30 | 932.92 | 151.26% | 226.85 | 221.90 | -2.18% |
| Barbados | 23.33 | 53.67 | 130.05% | 8.13 | 10.81 | 32.96% | 516.77 | 1241.47 | 140.24% | 194.50 | 255.64 | 31.43% |
| Belize | 3.41 | 16.87 | 394.72% | 3.70 | 6.07 | 64.05% | 88.76 | 479.85 | 440.62% | 92.62 | 156.40 | 68.86% |
| Bermuda | 7.60 | 11.75 | 54.61% | 12.40 | 8.9 | -28.23% | 184.66 | 250.18 | 35.48% | 291.06 | 202.71 | -30.35% |
| Cuba | 572.45 | 1,430.14 | 149.83% | 5.60 | 7.51 | 34.11% | 14581.13 | 34293.95 | 135.19% | 141.80 | 186.35 | 31.42% |
| Dominica | 5.27 | 8.45 | 60.34% | 7.43 | 9.3 | 25.17% | 120.29 | 192.88 | 60.35% | 180.96 | 215.64 | 19.16% |
| Dominican Republic | 83.97 | 511.63 | 509.30% | 2.25 | 5.48 | 143.56% | 2440.94 | 14237.10 | 483.26% | 59.14 | 145.53 | 146.08% |
| Grenada | 4.08 | 10.97 | 168.87% | 5.87 | 9.73 | 65.76% | 101.28 | 289.21 | 185.55% | 155.27 | 242.32 | 56.06% |
| Guyana | 16.54 | 40.03 | 142.02% | 4.38 | 6.31 | 44.06% | 465.83 | 1138.92 | 144.49% | 112.92 | 163.84 | 45.09% |
| Haiti | 73.71 | 186.59 | 153.14% | 2.26 | 2.7 | 19.47% | 2104.67 | 5223.47 | 148.18% | 58.25 | 68.33 | 17.30% |
| Jamaica | 68.86 | 231.13 | 235.65% | 3.91 | 7.71 | 97.19% | 1608.90 | 5529.35 | 243.67% | 93.90 | 185.21 | 97.24% |
| Puerto Rico | 274.35 | 539.34 | 96.59% | 7.64 | 7.65 | 0.13% | 6608.59 | 11805.91 | 78.64% | 184.28 | 188.19 | 2.12% |
| Saint Kitts and Nevis | 3.63 | 6.16 | 69.70% | 9.83 | 9.38 | -4.58% | 86.15 | 164.05 | 90.42% | 245.59 | 224.90 | -8.42% |
| Saint Lucia | 4.69 | 14.1 | 200.64% | 5.46 | 6.56 | 20.15% | 120.50 | 360.87 | 199.48% | 137.37 | 163.38 | 18.93% |
| Saint Vincent and the Grenadines | 3.18 | 9.58 | 201.26% | 4.48 | 7.11 | 58.71% | 80.05 | 245.16 | 206.26% | 111.71 | 176.90 | 58.36% |
| Suriname | 9.97 | 32.56 | 226.58% | 3.83 | 5.4 | 40.99% | 270.21 | 854.64 | 216.29% | 97.36 | 135.53 | 39.21% |
| Trinidad and Tobago | 57.59 | 139.04 | 141.43% | 7.05 | 7.4 | 4.96% | 1476.35 | 3426.15 | 132.07% | 171.46 | 179.89 | 4.92% |
| United States Virgin Islands | 6.48 | 23.12 | 256.79% | 7.95 | 12.52 | 57.48% | 171.15 | 535.57 | 212.92% | 190.50 | 294.39 | 54.54% |
| Colombia | 640.96 | 2,286.74 | 256.77% | 3.78 | 4.29 | 13.49% | 16911.63 | 54277.89 | 220.95% | 90.43 | 102.28 | 13.10% |
| Costa Rica | 76.40 | 335.62 | 339.29% | 4.46 | 6.53 | 46.41% | 1924.43 | 8016.10 | 316.54% | 106.00 | 153.43 | 44.75% |
| El Salvador | 78.89 | 275.68 | 249.45% | 2.66 | 4.62 | 73.68% | 2167.72 | 6602.50 | 204.58% | 69.43 | 112.93 | 62.65% |
| Guatemala | 84.27 | 404.62 | 380.15% | 2.35 | 3.73 | 58.72% | 2385.44 | 10814.71 | 353.36% | 58.22 | 92.27 | 58.49% |
| Honduras | 57.59 | 392.15 | 580.93% | 2.83 | 6.71 | 137.10% | 1581.17 | 9947.65 | 529.13% | 70.76 | 157.46 | 122.53% |
| Mexico | 1805.62 | 6,587.50 | 264.83% | 4.34 | 5.68 | 30.88% | 47886.23 | 168702.09 | 252.30% | 104.62 | 139.18 | 33.03% |
| Nicaragua | 31.43 | 196.75 | 525.99% | 2.08 | 4.73 | 127.40% | 861.63 | 4839.19 | 461.63% | 51.68 | 105.43 | 104.01% |
| Panama | 28.71 | 182.24 | 534.76% | 1.96 | 4.39 | 123.98% | 728.04 | 4404.31 | 504.95% | 47.17 | 106.02 | 124.76% |
| Venezuela (Bolivarian Republic of) | 448.14 | 1,472.34 | 228.54% | 4.71 | 5.08 | 7.86% | 12148.10 | 37064.89 | 205.11% | 117.61 | 123.08 | 4.65% |
| Brazil | 4042.45 | 15,564.89 | 285.04% | 4.69 | 6.62 | 41.15% | 107871.60 | 380858.91 | 253.07% | 113.75 | 156.95 | 37.98% |
| Paraguay | 72.98 | 308.16 | 322.25% | 3.36 | 5.64 | 67.86% | 1873.08 | 7673.30 | 309.66% | 81.51 | 133.69 | 64.02% |
| Afghanistan | 288.00 | 769.37 | 167.14% | 3.94 | 6.26 | 58.88% | 8303.44 | 23546.22 | 183.57% | 106.83 | 159.54 | 49.34% |
| Algeria | 338.75 | 1,427.23 | 321.32% | 2.98 | 4.61 | 54.70% | 8951.16 | 34889.06 | 289.77% | 68.82 | 100.88 | 46.59% |
| Bahrain | 12.63 | 62.99 | 398.73% | 8.09 | 8.04 | -0.62% | 354.31 | 1769.26 | 399.35% | 181.29 | 164.69 | -9.16% |
| Egypt | 1475.51 | 6,077.75 | 311.91% | 4.96 | 9.27 | 86.90% | 43007.96 | 175507.84 | 308.08% | 130.79 | 240.95 | 84.23% |
| Iran (Islamic Republic of) | 786.75 | 3,434.27 | 336.51% | 3.17 | 4.95 | 56.15% | 22094.07 | 85559.67 | 287.25% | 75.36 | 112.71 | 49.56% |
| Iraq | 337.88 | 1,303.92 | 285.91% | 4.43 | 5.96 | 34.54% | 9305.82 | 35903.97 | 285.82% | 111.91 | 143.58 | 28.30% |
| Jordan | 72.64 | 445.4 | 513.16% | 5.74 | 7.48 | 30.31% | 2028.05 | 11749.68 | 479.36% | 135.79 | 168.16 | 23.84% |
| Kuwait | 29.36 | 147.58 | 402.66% | 5.20 | 6.54 | 25.77% | 865.11 | 3870.31 | 347.38% | 122.11 | 141.68 | 16.03% |
| Lebanon | 114.03 | 430.29 | 277.35% | 5.19 | 8.35 | 60.89% | 2859.81 | 9693.71 | 238.96% | 118.83 | 188.65 | 58.76% |
| Libya | 96.82 | 366.8 | 278.85% | 5.27 | 7.51 | 42.50% | 2667.40 | 9905.00 | 271.34% | 131.46 | 183.05 | 39.24% |
| Morocco | 276.03 | 1,271.28 | 360.56% | 2.10 | 4.18 | 99.05% | 6896.04 | 31099.34 | 350.97% | 49.26 | 94.10 | 91.03% |
| Oman | 15.99 | 106.28 | 564.67% | 2.40 | 6.87 | 186.25% | 482.90 | 3157.86 | 553.94% | 61.06 | 153.97 | 152.16% |
| Palestine | 41.04 | 156.63 | 281.65% | 4.75 | 6.89 | 45.05% | 1107.74 | 4294.32 | 287.66% | 120.29 | 165.08 | 37.24% |
| Qatar | 9.84 | 89.92 | 813.82% | 11.40 | 16.13 | 41.49% | 292.69 | 2699.02 | 822.14% | 242.93 | 291.55 | 20.01% |
| Saudi Arabia | 199.63 | 1,176.87 | 489.53% | 3.57 | 7.14 | 100.00% | 5632.89 | 36554.20 | 548.94% | 85.77 | 169.13 | 97.19% |
| Sudan | 169.99 | 805.92 | 374.10% | 1.84 | 4.52 | 145.65% | 4739.25 | 21967.78 | 363.53% | 47.47 | 108.41 | 128.38% |
| Syrian Arab Republic | 200.85 | 549.41 | 173.54% | 3.77 | 4.64 | 23.08% | 6027.14 | 14841.55 | 146.25% | 98.67 | 110.92 | 12.42% |
| Tunisia | 125.23 | 504.17 | 302.60% | 2.59 | 4.1 | 58.30% | 3136.11 | 11848.00 | 277.79% | 59.11 | 91.32 | 54.49% |
| Turkey | 2037.14 | 6,020.15 | 195.52% | 5.70 | 6.92 | 21.40% | 56641.92 | 146406.44 | 158.48% | 144.67 | 161.85 | 11.88% |
| United Arab Emirates | 41.65 | 626.52 | 1404.25% | 9.89 | 15.24 | 54.10% | 1452.15 | 22934.50 | 1479.35% | 235.58 | 358.50 | 52.18% |
| Yemen | 61.62 | 301.51 | 389.31% | 1.26 | 2.32 | 84.13% | 1768.55 | 8451.08 | 377.85% | 32.29 | 58.24 | 80.37% |
| Bangladesh | 201.80 | 1,518.84 | 652.65% | 0.44 | 1.16 | 163.64% | 5728.99 | 41538.54 | 625.06% | 11.23 | 30.19 | 168.83% |
| Bhutan | 2.88 | 16.34 | 467.36% | 1.15 | 2.95 | 156.52% | 85.50 | 428.06 | 400.65% | 30.31 | 72.49 | 139.16% |
| India | 3213.58 | 23,548.08 | 632.77% | 0.74 | 2.1 | 183.78% | 91851.48 | 626180.46 | 581.73% | 18.25 | 52.05 | 185.21% |
| Nepal | 48.86 | 441.66 | 803.93% | 0.52 | 2 | 284.62% | 1436.41 | 11734.91 | 716.96% | 13.46 | 49.50 | 267.76% |
| Pakistan | 939.49 | 5,077.73 | 440.48% | 1.70 | 4.88 | 187.06% | 24095.04 | 134933.58 | 460.01% | 41.18 | 116.22 | 182.22% |
| China | 30067.26 | 100441.58 | 234.06% | 3.51 | 4.99 | 42.17% | 885448.10 | 2669799.24 | 201.52% | 93.74 | 128.06 | 36.61% |
| Democratic People's Republic of Korea | 344.13 | 646.92 | 87.99% | 2.06 | 1.99 | -3.40% | 10561.91 | 18163.33 | 71.97% | 56.92 | 54.27 | -4.66% |
| Taiwan (Province of China) | 625.21 | 2,684.75 | 329.42% | 3.87 | 6.8 | 75.71% | 18066.42 | 69910.28 | 286.96% | 103.10 | 181.29 | 75.84% |
| American Samoa | 2.45 | 7.01 | 186.12% | 10.80 | 14.5 | 34.26% | 74.61 | 200.51 | 168.74% | 281.65 | 383.02 | 35.99% |
| Cook Islands | 1.33 | 2.93 | 120.30% | 10.67 | 11.95 | 12.00% | 37.63 | 73.51 | 95.35% | 276.36 | 299.85 | 8.50% |
| Fiji | 28.27 | 84.8 | 199.96% | 7.76 | 11.72 | 51.03% | 882.65 | 2420.60 | 174.24% | 205.91 | 291.36 | 41.50% |
| Guam | 5.43 | 17.03 | 213.63% | 7.60 | 8.92 | 17.37% | 161.97 | 468.93 | 189.52% | 183.77 | 243.01 | 32.24% |
| Kiribati | 3.79 | 7.61 | 100.79% | 9.38 | 10.06 | 7.25% | 126.49 | 253.67 | 100.55% | 282.81 | 292.63 | 3.47% |
| Marshall Islands | 0.84 | 3.06 | 264.29% | 5.10 | 8.16 | 60.00% | 26.20 | 100.47 | 283.47% | 140.82 | 232.04 | 64.78% |
| Micronesia (Federated States of) | 4.16 | 9.46 | 127.40% | 8.55 | 12.1 | 41.52% | 134.09 | 302.90 | 125.89% | 254.61 | 347.05 | 36.31% |
| Nauru | 0.43 | 0.57 | 32.56% | 10.59 | 12.28 | 15.96% | 14.69 | 19.98 | 36.01% | 294.46 | 342.30 | 16.25% |
| Niue | 0.18 | 0.24 | 33.33% | 8.25 | 10.95 | 32.73% | 4.63 | 6.20 | 33.91% | 221.75 | 284.70 | 28.39% |
| Northern Mariana Islands | 2.10 | 7.27 | 246.19% | 11.67 | 13.58 | 16.37% | 73.72 | 217.34 | 194.82% | 299.18 | 350.07 | 17.01% |
| Palau | 0.82 | 2.32 | 182.93% | 8.64 | 10.78 | 24.77% | 24.08 | 69.21 | 187.42% | 228.84 | 282.75 | 23.56% |
| Papua New Guinea | 57.76 | 203.55 | 252.41% | 2.86 | 3.81 | 33.22% | 1827.76 | 6609.18 | 261.60% | 82.29 | 110.78 | 34.62% |
| Samoa | 7.10 | 12.11 | 70.56% | 7.91 | 8.08 | 2.15% | 209.49 | 357.21 | 70.51% | 219.09 | 221.91 | 1.29% |
| Solomon Islands | 6.46 | 35.36 | 447.37% | 4.04 | 9.8 | 142.57% | 227.94 | 1227.68 | 438.60% | 127.25 | 305.91 | 140.40% |
| Tokelau | 0.08 | 0.12 | 50.00% | 6.17 | 8.78 | 42.30% | 2.26 | 3.29 | 45.58% | 176.43 | 239.60 | 35.80% |
| Tonga | 6.09 | 10.64 | 74.71% | 10.22 | 13.23 | 29.45% | 190.17 | 312.91 | 64.54% | 303.74 | 378.74 | 24.69% |
| Tuvalu | 0.41 | 0.85 | 107.32% | 5.86 | 8.2 | 39.93% | 12.48 | 25.14 | 101.44% | 166.78 | 228.50 | 37.01% |
| Vanuatu | 2.77 | 11.76 | 324.55% | 4.15 | 6.62 | 59.52% | 86.77 | 365.27 | 320.96% | 113.80 | 186.55 | 63.93% |
| Cambodia | 53.17 | 338.75 | 537.11% | 1.12 | 2.78 | 148.21% | 1679.78 | 9848.58 | 486.30% | 31.72 | 73.79 | 132.63% |
| Indonesia | 1157.76 | 8,725.90 | 653.69% | 1.11 | 3.77 | 239.64% | 36984.39 | 268005.76 | 624.65% | 31.17 | 104.88 | 236.48% |
| Lao People's Democratic Republic | 35.96 | 178.09 | 395.24% | 1.65 | 3.86 | 133.94% | 1111.57 | 5459.18 | 391.12% | 46.85 | 106.28 | 126.85% |
| Malaysia | 343.57 | 1,959.68 | 470.39% | 3.64 | 7.38 | 102.75% | 10252.82 | 53732.54 | 424.08% | 98.64 | 186.12 | 88.69% |
| Maldives | 1.39 | 8.57 | 516.55% | 1.42 | 2.75 | 93.66% | 43.82 | 255.80 | 483.75% | 39.27 | 70.87 | 80.47% |
| Mauritius | 24.10 | 109.96 | 356.27% | 3.29 | 6.2 | 88.45% | 674.33 | 2875.68 | 326.45% | 85.22 | 156.75 | 83.94% |
| Myanmar | 350.49 | 1,651.64 | 371.24% | 1.43 | 3.46 | 141.96% | 10835.60 | 48062.94 | 343.57% | 40.94 | 93.29 | 127.87% |
| Philippines | 873.91 | 3,614.05 | 313.55% | 2.83 | 4.49 | 58.66% | 27302.56 | 108568.23 | 297.65% | 77.76 | 122.12 | 57.05% |
| Seychelles | 2.96 | 9.72 | 228.38% | 5.30 | 8.39 | 58.30% | 86.52 | 288.21 | 233.11% | 155.17 | 233.77 | 50.65% |
| Sri Lanka | 222.60 | 989.57 | 344.55% | 2.08 | 3.94 | 89.42% | 6336.47 | 24777.70 | 291.03% | 53.10 | 93.89 | 76.82% |
| Thailand | 1047.81 | 6,955.59 | 563.82% | 2.76 | 6.69 | 142.39% | 32953.03 | 191848.16 | 482.19% | 77.98 | 183.24 | 134.98% |
| Timor-Leste | 2.76 | 12.79 | 363.41% | 0.89 | 1.55 | 74.16% | 91.23 | 364.06 | 299.06% | 24.69 | 41.77 | 69.18% |
| Viet Nam | 290.77 | 2,543.36 | 774.70% | 0.73 | 2.74 | 275.34% | 7883.72 | 69391.14 | 780.18% | 18.74 | 68.39 | 264.94% |
| Angola | 44.02 | 322.52 | 632.67% | 1.13 | 2.93 | 159.29% | 1317.10 | 9373.95 | 611.71% | 29.20 | 73.34 | 151.16% |
| Central African Republic | 18.89 | 41.97 | 122.18% | 1.57 | 1.92 | 22.29% | 561.11 | 1258.35 | 124.26% | 41.65 | 49.99 | 20.02% |
| Congo | 46.30 | 150.48 | 225.01% | 4.28 | 5.99 | 39.95% | 1308.96 | 4230.80 | 223.22% | 110.86 | 147.06 | 32.65% |
| Democratic Republic of the Congo | 421.62 | 807.01 | 91.41% | 2.72 | 2.35 | -13.60% | 11832.64 | 22484.78 | 90.02% | 67.15 | 57.62 | -14.19% |
| Equatorial Guinea | 2.62 | 34.26 | 1207.63% | 1.31 | 7.86 | 500.00% | 75.63 | 911.68 | 1105.45% | 34.18 | 177.96 | 420.66% |
| Gabon | 23.16 | 85.07 | 267.31% | 4.09 | 8.42 | 105.87% | 638.64 | 2287.09 | 258.12% | 105.86 | 201.75 | 90.58% |
| Burundi | 43.89 | 92.67 | 111.14% | 1.88 | 2.03 | 7.98% | 1258.46 | 2696.97 | 114.31% | 50.11 | 51.68 | 3.13% |
| Comoros | 6.01 | 19.37 | 222.30% | 2.75 | 4.04 | 46.91% | 162.83 | 519.11 | 218.80% | 69.58 | 101.16 | 45.39% |
| Djibouti | 1.88 | 22.95 | 1120.74% | 1.35 | 3.88 | 187.41% | 57.86 | 673.93 | 1064.76% | 34.11 | 96.93 | 184.17% |
| Eritrea | 13.23 | 92.47 | 598.94% | 1.38 | 3.79 | 174.64% | 395.34 | 2565.98 | 549.06% | 35.00 | 89.99 | 157.11% |
| Ethiopia | 179.54 | 664.24 | 269.97% | 0.89 | 1.67 | 87.64% | 5541.09 | 18342.73 | 231.03% | 23.66 | 40.67 | 71.89% |
| Kenya | 160.17 | 1,096.82 | 584.78% | 1.94 | 5.02 | 158.76% | 4592.35 | 31390.65 | 583.54% | 50.42 | 126.29 | 150.48% |
| Madagascar | 80.56 | 297.65 | 269.48% | 1.58 | 2.73 | 72.78% | 2294.73 | 8749.16 | 281.27% | 40.63 | 69.35 | 70.69% |
| Malawi | 89.01 | 412.67 | 363.62% | 2.26 | 5.83 | 157.96% | 2697.73 | 11498.17 | 326.22% | 60.62 | 145.60 | 140.18% |
| Mozambique | 45.66 | 299.86 | 556.72% | 0.81 | 2.88 | 255.56% | 1218.42 | 8257.95 | 577.76% | 19.08 | 70.03 | 267.03% |
| Rwanda | 73.15 | 216.62 | 196.13% | 2.50 | 3.74 | 49.60% | 2120.09 | 5895.88 | 178.10% | 66.20 | 90.02 | 35.98% |
| Somalia | 33.51 | 82.8 | 147.09% | 1.28 | 1.26 | -1.56% | 1055.07 | 2512.78 | 138.16% | 34.37 | 32.95 | -4.13% |
| South Sudan | 70.44 | 158.88 | 125.55% | 3.00 | 4.26 | 42.00% | 1960.08 | 4470.00 | 128.05% | 76.42 | 104.31 | 36.50% |
| Uganda | 98.73 | 742.81 | 652.37% | 1.54 | 5.37 | 248.70% | 2735.16 | 20936.07 | 665.44% | 39.03 | 135.22 | 246.45% |
| United Republic of Tanzania | 330.18 | 1,193.15 | 261.36% | 3.09 | 5.01 | 62.14% | 9155.86 | 32767.72 | 257.89% | 77.30 | 123.31 | 59.52% |
| Zambia | 92.08 | 370.67 | 302.55% | 3.16 | 5.6 | 77.22% | 2735.37 | 10936.70 | 299.83% | 83.68 | 144.48 | 72.66% |
| Botswana | 20.24 | 119.78 | 491.80% | 3.65 | 9.41 | 157.81% | 545.69 | 3250.07 | 495.59% | 89.62 | 221.63 | 147.30% |
| Eswatini | 25.03 | 90.92 | 263.24% | 9.09 | 16.28 | 79.10% | 684.90 | 2591.79 | 278.42% | 218.31 | 406.77 | 86.33% |
| Lesotho | 32.38 | 131.18 | 305.13% | 3.38 | 10.69 | 216.27% | 847.84 | 3604.54 | 325.14% | 82.11 | 264.63 | 222.29% |
| Namibia | 14.62 | 58.84 | 302.46% | 2.10 | 4.46 | 112.38% | 372.86 | 1443.02 | 287.01% | 50.67 | 102.80 | 102.88% |
| South Africa | 1572.43 | 4,194.44 | 166.75% | 7.69 | 9.85 | 28.09% | 43144.16 | 105257.18 | 143.97% | 193.17 | 225.44 | 16.71% |
| Zimbabwe | 205.68 | 549.44 | 167.13% | 5.11 | 8.03 | 57.14% | 5712.00 | 15498.34 | 171.33% | 128.24 | 203.62 | 58.78% |
| Benin | 37.74 | 195.41 | 417.78% | 1.91 | 4.05 | 112.04% | 1047.47 | 5546.13 | 429.48% | 50.43 | 103.72 | 105.67% |
| Burkina Faso | 41.83 | 235.07 | 461.97% | 1.00 | 2.76 | 176.00% | 1075.30 | 6144.59 | 471.43% | 23.44 | 64.82 | 176.54% |
| Cabo Verde | 5.05 | 39.5 | 682.18% | 2.19 | 9.35 | 326.94% | 116.39 | 1027.57 | 782.87% | 52.08 | 229.61 | 340.88% |
| Cameroon | 149.28 | 629.33 | 321.58% | 3.51 | 5.71 | 62.68% | 3977.24 | 16710.02 | 320.14% | 83.56 | 132.92 | 59.07% |
| Chad | 21.52 | 101.21 | 370.31% | 0.77 | 1.85 | 140.26% | 569.21 | 2815.16 | 394.57% | 19.54 | 46.19 | 136.39% |
| Côte d'Ivoire | 101.87 | 389.57 | 282.42% | 2.53 | 3.74 | 47.83% | 3098.16 | 11421.92 | 268.67% | 64.68 | 93.29 | 44.23% |
| Gambia | 11.03 | 74.16 | 572.35% | 2.91 | 7.28 | 150.17% | 351.19 | 2344.63 | 567.62% | 82.69 | 209.13 | 152.91% |
| Ghana | 109.97 | 911.92 | 729.24% | 1.74 | 6.02 | 245.98% | 3125.47 | 23713.03 | 658.70% | 44.90 | 142.07 | 216.41% |
| Guinea | 89.00 | 255.87 | 187.49% | 2.69 | 4.63 | 72.12% | 2444.47 | 7268.94 | 197.36% | 69.54 | 120.39 | 73.12% |
| Guinea-Bissau | 7.34 | 22.38 | 204.90% | 1.82 | 3.18 | 74.73% | 209.34 | 641.43 | 206.41% | 47.43 | 80.76 | 70.27% |
| Liberia | 30.69 | 84.15 | 174.19% | 2.75 | 4.11 | 49.45% | 841.82 | 2478.35 | 194.40% | 72.54 | 107.49 | 48.18% |
| Mali | 64.08 | 280.71 | 338.06% | 1.51 | 3.21 | 112.58% | 1832.64 | 8090.63 | 341.47% | 39.96 | 84.38 | 111.16% |
| Mauritania | 36.35 | 101.92 | 180.39% | 3.71 | 5.2 | 40.16% | 952.40 | 2496.77 | 162.16% | 91.60 | 116.60 | 27.29% |
| Niger | 23.29 | 108.65 | 366.51% | 0.87 | 1.45 | 66.67% | 647.31 | 2950.38 | 355.79% | 21.44 | 34.87 | 62.64% |
| Nigeria | 430.43 | 1,920.40 | 346.16% | 1.02 | 2.44 | 139.22% | 10971.00 | 47821.60 | 335.89% | 24.01 | 54.71 | 127.86% |
| Sao Tome and Principe | 1.53 | 5.87 | 283.66% | 2.40 | 5.9 | 145.83% | 39.15 | 156.59 | 299.97% | 57.59 | 139.73 | 142.63% |
| Senegal | 55.80 | 238.87 | 328.08% | 1.80 | 3.36 | 86.67% | 1438.62 | 6041.85 | 319.98% | 42.88 | 77.70 | 81.20% |
| Sierra Leone | 22.58 | 82.22 | 264.13% | 1.21 | 2.35 | 94.21% | 587.43 | 2259.92 | 284.71% | 29.89 | 59.17 | 97.96% |
| Togo | 19.90 | 114.44 | 475.08% | 1.66 | 3.28 | 97.59% | 553.69 | 3167.82 | 472.13% | 41.09 | 79.31 | 93.02% |

Abbreviations: ASDR, age-standardized DALY rate; ASMR, age-standardized mortality rate; DALYs, disability-adjusted life-years.

**Supplementary Table 2** Time trend analysis of ASMR for cancer attributable to high body mass index from 1990 to 2019 by country

| Country | Trend 1 |  | Trend 2 |  | Trend 3 |  | AAPC |
| --- | --- | --- | --- | --- | --- | --- | --- |
|  | Period | APC | Period | APC | Period | APC |  |
| Armenia | 1990-2001 | 0.6 (0.2, 1.0) | 2001-2011 | 6.3 (5.7, 6.9) | 2011-2019 | 0.6 (0, 1.3) | 2.5 (2.2, 2.8) |
| Azerbaijan | 1990-2001 | -0.7 (-1.1, -0.3) | 2001-2012 | 4.3 (3.9, 4.7) | 2012-2019 | 1.0 (0.2, 1.7) | 1.6 (1.3, 1.8) |
| Georgia | 1990-1996 | -5.1 (-6.6, -3.6) | 1996-2002 | 3.7 (1.5, 5.9) | 2002-2019 | 2.0 (1.6, 2.3) | 0.8 (0.3, 1.4) |
| Kazakhstan | 1990-1995 | 2.5 (1.5, 3.5) | 1995-2000 | -3.2 (-4.5, -1.9) | 2000-2019 | -0.5 (-0.6, -0.4) | -0.5 (-0.8, -0.2) |
| Kyrgyzstan | 1990-1994 | 2.6 (1.1, 4.1) | 1994-1999 | -3.4 (-4.8, -2.0) | 1999-2019 | 0.2 (0.1, 0.3) | -0.1 (-0.4, 0.2) |
| Mongolia | 1990-1998 | 2.3 (1.9, 2.6) | 1998-2009 | 3.2 (3.0, 3.5) | 2009-2019 | 0 (-0.3, 0.2) | 1.8 (1.7, 2.0) |
| Tajikistan | 1990-1994 | 0.7 (-1.7, 3.1) | 1994-1998 | -5.5 (-9.0, -1.8) | 1998-2019 | 2.2 (2.0, 2.4) | 0.9 (0.3, 1.5) |
| Turkmenistan | 1990-1994 | -3.0 (-6.1, 0.2) | 1994-2000 | -7.8 (-9.9, -5.7) | 2000-2019 | 1.6 (1.3, 1.9) | -1.1 (-1.7, -0.4) |
| Uzbekistan | 1990-1995 | 1.8 (0.4, 2.2) | 1995-2003 | -1.7 (-2.5, -0.9) | 2003-2019 | 1.7 (1.5, 1.9) | 0.8 (0.4, 1.1) |
| Albania | 1990-1994 | -2.2 (-4.7, 0.3) | 1994-2011 | 0.7 (0.4, 1.0) | 2011-2019 | 2.1 (1.2, 3.0) | 0.7 (0.2, 1.1) |
| Bosnia and Herzegovina | 1990-1997 | 0.3 (-0.2, 0.8) | 1997-2005 | 5.2 (4.7, 5.8) | 2005-2019 | 0.7 (0.5, 0.9) | 1.8 (1.6, 2.0) |
| Bulgaria | 1990-2000 | -2.8 (-3.2, -2.3) | 2000-2007 | 7.1 (6.1, 8.1) | 2007-2019 | 0.7 (0.4, 1.0) | 1.0 (0.7, 1.3) |
| Croatia | 1990-2001 | 0.5 (0.1, 0.9) | 2001-2011 | 3.1 (2.5, 3.6) | 2011-2019 | -0.6 (-1.2, 0.1) | 1.1 (0.8, 1.4) |
| Czechia | 1990-2006 | 0.7 (0.4, 0.9) | 2006-2016 | -1.6 (-2.2, -1.1) | 2016-2019 | 0.4 (-2.6, 3.5) | -0.2 (-0.5, 0.2) |
| Hungary | 1990-2008 | 0.2 (0, 0.3) | 2008-2014 | -2.3 (-3.4, -1.3) | 2014-2019 | 0 (-1.1, 1.1) | -0.4 (-0.7, -0.1) |
| Montenegro | 1990-2000 | 0.8 (0.5, 1.1) | 2000-2008 | 1.9 (1.4, 2.4) | 2008-2019 | 0.8 (0.5, 1.0) | 1.1 (0.9, 1.3) |
| North Macedonia | 1990-1997 | 2.0 (1.7, 2.4) | 1997-2007 | 2.8 (2.5, 3.0) | 2007-2019 | 0.3 (0.1, 0.4) | 1.6 (1.4, 1.7) |
| Poland | 1990-1996 | 0.6 (0.1, 1.0) | 1996-1999 | 2.3 (-0.3, 5.0) | 1999-2019 | 0.4 (0.4, 0.5) | 0.7 (0.4, 0.9) |
| Romania | 1990-1996 | 3.7 (2.6, 4.8) | 1996-1999 | -1.5 (-7.4, 4.7) | 1999-2019 | 2.1 (2.0, 2.3) | 2.1 (1.4, 2.7) |
| Serbia | 1990-2002 | 1.0 (0.7, 1.4) | 2002-2008 | 2.8 (2.5, 4.1) | 2008-2019 | 0.5 (0.1, 0.9) | 1.2 (0.9, 1.5) |
| Slovakia | 1990-1999 | 2.7 (2.1, 3.2) | 1999-2003 | -1.7 (-4.7, 1.4) | 2003-2019 | 0.4 (0.1, 0.6) | 0.8 (0.3, 1.2) |
| Slovenia | 1990-2001 | 2.0 (1.7, 2.3) | 2001-2009 | 0.3 (-0.3, 1.0) | 2009-2019 | -1.0 (-1.4, -0.6) | 0.5 (0.3, 0.7) |
| Belarus | 1990-1993 | 5.9 (1.8, 10.1) | 1993-2002 | 1.7 (0.8, 2.6) | 2002-2019 | 0.4 (0.1, 0.6) | 1.3 (0.8, 1.8) |
| Estonia | 1990-1999 | 1.4 (0.6, 2.1) | 1999-2007 | 3.0 (1.8, 4.1) | 2007-2019 | 0.4 (-0.2, 0.9) | 1.4 (0.9, 1.8) |
| Latvia | 1990-2004 | 1.6 (1.0, 2.3) | 2004-2007 | 6.0 (-7.5, 21.5) | 2007-2019 | -0.4 (-1.2, 0.4) | 1.2 (-0.2, 2.6) |
| Lithuania | 1990-2004 | 2.0 (1.6, 2.5) | 2004-2007 | 4.3 (-5.4, 14.9) | 2007-2019 | -0.4 (-1.0, 0.1) | 1.2 (0.2, 2.2) |
| Republic of Moldova | 1990-2000 | -1.0 (-2.1, 0) | 2000-2015 | 3.0 (2.3, 3.7) | 2015-2019 | -2.6 (-6.9, 1.8) | 0.8 (0, 1.6) |
| Russian Federation | 1990-1994 | 6.7 (3.7, 9.8) | 1994-1997 | -3.3 (-11.8, 5.9) | 1997-2019 | 0.8 (0.6, 1.1) | 1.2 (0.2, 2.2) |
| Ukraine | 1990-1994 | 3.3 (-0.1, 6.9) | 1994-2006 | -1.2 (-1.9, 6.9) | 2006-2019 | 1.4 (0.9, 2.0) | 0.6 (0, 1.2) |
| Australia | 1990-1996 | 1.4 (1.0, 1.7) | 1996-2017 | 0.1 (0.1, 0.2) | 2017-2019 | 0.8 (-1.2, 2.9) | 0.4 (0.3, 0.6) |
| New Zealand | 1990-1993 | 1.5 (0, 3.0) | 1993-2001 | 0.1 (-0.3, 0.5) | 2001-2019 | 0.3 (0.2, 0.4) | 0.4 (0.2, 0.6) |
| Brunei Darussalam | 1900-1996 | -0.9 (-1.8, 0.1) | 1996-2007 | 4.8 (4.4, 5.3) | 2007-2019 | 1.4 (1.1, 1.8) | 2.2 (1.9, 2.5) |
| Japan | 1990-1998 | 1.7 (1.5, 2.0) | 1998-2017 | -1.4 (-1.5, -1.4) | 2017-2019 | 0.6 (-1.7, 2.8) | -0.4 (-0.6, -0.3) |
| Republic of Korea | 1990-1996 | 1.3 (0.5, 2.0) | 1996-2000 | 7.8 (5.5, 10.1) | 2000-2019 | -0.7 (-0.8, -0.6) | 0.9 (0.5, 1.2) |
| Singapore | 1990-1997 | 3.5 (3.1, 3.9) | 1997-2010 | 0.9 (0.7, 1.0) | 2010-2019 | -1.0 (-1.0, -0.4) | 1.0 (0.9, 1.1) |
| Canada | 1990-1998 | 1.4 (1.2, 1.5) | 1998-2008 | 0.9 (0.7, 1.0) | 2008-2019 | 0 (-0.1, 0.1) | 0.7 (0.6, 0.7) |
| Greenland | 1990-2000 | 2.9 (2.5, 3.2) | 2000-2004 | -1.7 (-3.9, 0.5) | 2004-2019 | 0 (-0.1, 0.2) | 0.8 (0.4, 1.1) |
| United States of America | 1990-1995 | 2.0 (1.8, 2.3) | 1995-2002 | 1.1 (0.9, 1.3) | 2002-2019 | 0.3 (0.2, 0.3) | 0.8 (0.7, 0.8) |
| Argentina | 1990-2002 | 1.9 (1.7, 2.0) | 2002-2014 | -0.3 (-0.4, -0.1) | 2014-2019 | 1.5 (0.9, 2.1) | 0.9 (0.8, 1.0) |
| Chile | 1990-2000 | 1.4 (1.2, 1.6) | 2000-2016 | -1.0 (-1.2, -0.9) | 2016-2019 | 0.8 (-0.6, 2.3) | 0 (-0.2, 0.2) |
| Uruguay | 1990-2003 | 1.6 (1.4, 1.8) | 2003-2013 | -0.3 (-0.7, 0) | 2013-2019 | 1.1 (0.5, 1.7) | 0.8 (0.7, 1.0) |
| Andorra | 1990-1993 | -0.4 (-1.1, 0.2) | 1993-2000 | -1.0 (-1.2, -0.8) | 2000-2019 | 0.3 (0.3, 0.4) | -0.1 (-0.2, 0) |
| Austria | 1990-1992 | 1.2 (-1.1, 3.5) | 1992-1997 | -0.1 (-0.8, 0.6) | 1997-2019 | -0.7 (-0.8, -0.7) | -0.5 (-0.7, -0.3) |
| Belgium | 1990-1994 | 1.6 (0.2, 3.0) | 1994-2006 | -0.1 (-0.4, 0.1) | 2006-2019 | 0.3 (0.1, 0.5) | 0.3 (0.1, 0.5) |
| Cyprus | 1990-2002 | 1.1 (0.7, 1.4) | 2002-2007 | 3.4 (1.5, 5.3) | 2007-2019 | -0.5 (-0.9, -0.2) | 0.8 (0.4, 1.2) |
| Denmark | 1990-1996 | 3.4 (3.0, 3.8) | 1996-2010 | 0.3 (0.2, 0.4) | 2010-2019 | -0.9 (-1.1, -0.7) | 0.6 (0.5, 0.7) |
| Finland | 1990-2004 | 0.5 (0.4, 0.6) | 2004-2013 | 0.1 (0, 0.3) | 2013-2019 | -0.4 (-0.7, -0.2 | 0.2 (0.1, 0.3) |
| France | 1990-1999 | 0.5 (0.3, 0.7) | 1999-2011 | -0.3 (-0.5, -0.2) | 2011-2019 | -0.5 (-0.7, -0.3) | -0.1 (-0.2, 0) |
| Germany | 1990-2004 | 1.6 (1.5, 1.8) | 2004-2013 | -1.5 (-1.8, -1.2) | 2013-2019 | 0.1 (-0.4, 0.6) | 0.3 (0.2, 0.5) |
| Greece | 1990-2002 | 2.0 (1.9, 2.2) | 2002-2017 | -0.5 (-0.6, -0.3) | 2017-2019 | 1.0 (-1.4, 3.5) | 0.7 (0.5, 0.8) |
| Iceland | 1990-1994 | 0.6 (-0.8, 2.0) | 1994-2012 | -0.1 (-0.3, 0) | 2012-2019 | -0.8 (-1.4, -0.2) | -0.2 (-0.4, 0) |
| Ireland | 1990-2000 | 0.9 (0.8, 1.0) | 2000-2012 | 0 (-0.1, 0.2) | 2012-2019 | -0.3 (-0.5, -0.1) | 0.2 (0.2, 0.3) |
| Israel | 1990-1998 | 2.5 (2.2, 2.9) | 1998-2017 | -1.1 (-1.2, -0.9) | 2017-2019 | 1.2 (-2.0, 4.6) | 0.1 (-0.2, 0.3) |
| Italy | 1990-1994 | 1.5 (0.9, 2.2) | 1994-2000 | 0.5 (0.1, 1.0) | 2000-2019 | -0.6 (-0.7, -0.6) | -0.1 (-0.2, 0) |
| Luxembourg | 1990-2013 | -0.6 (-0.7, -0.6) | 2013-2016 | -3.6 (-5.3, -1.8) | 2016-2019 | -0.2 (-1.1, 0.7) | -0.9 (-1.1, 0.7) |
| Malta | 1990-2003 | 1.7 (1.6, 1.8) | 2003-2016 | -1.3 (-1.4, -1.2) | 2017-2019 | -0.2 (-1.0, 0.7) | 0.1 (0, 0.2) |
| Monaco | 1900-1996 | 1.1 (0.6, 1.7) | 3.5 (2.9, 4.0) | 1996-2003 | 0.2 (0.1, 0.3) | 2003-2019 | 1.2 (1.0, 1.3) |
| Netherlands | 1990-1992 | 2.0 (-1.1, 5.1) | 1992-2016 | 0.8 (0.7, 0.9) | 2016-2019 | 0 (-1.5, 1.5) | 0.8 (0.5, 1.0) |
| Norway | 1990-2001 | 0.9 (0.7, 1.0) | 2001-2013 | 0.5 (0.4, 0.7) | 2013-2019 | -1.0 (-1.4, -0.6) | 0.3 (0.2, 0.4) |
| Portugal | 1990-1997 | 2.7 (2.3, 3.0) | 1997-2008 | 0.5 (0.3, 0.7) | 2008-2019 | -0.6 (-0.8, -0.4) | 0.6 (0.5, 0.7) |
| San Marino | 1990-1999 | -0.2 (-0.4, 0) | 1999-2009 | 1.6 (1.4, 1.8) | 2009-2019 | -0.2 (-0.4, 0) | 0.4 (0.3, 0.5) |
| Spain | 1990-1995 | 2.2 (1.8, 2.6) | 1995-1999 | 1.3 (0.4, 2.3) | 1999-2019 | -0.1 (-0.2, -0.1) | 0.5 (0.3, 0.6) |
| Sweden | 1990-1996 | 0.6 (0.4, 0.9) | 1996-2009 | -0.1 (-0.2, 0) | 20009-2019 | 0.3 (0.2, 0.5) | 0.2 (0.1, 0.3) |
| Switzerland | 1990-1999 | 1.1 (0.7, 1.4) | 199-2006 | -0.8 (-1.5, -0.2) | 2006-2019 | 0.1 (-0.1, 0.3) | 0.2 (0, 0.4) |
| United Kingdom | 1990-2002 | 0.9 (0.8, 1.0) | 2002-2011 | 0.1 (-0.1, 0.2) | 2011-2019 | 0.5 (0.3, 0.6) | 0.5 (0.5, 0.6) |
| Bolivia (Plurinational State of) | 1990-2011 | 1.9 (1.8, 2.0) | 2011-2014 | 0.7 (-1.4, 2.8) | 2014-2019 | 2.6 (2.1, 3.1) | 1.9 (1.7, 2.1) |
| Ecuador | 1990-2001 | -0.6 (-1.0, -0.2) | 2001-2004 | 8.9 (2.0, 15.1) | 2004-2019 | 1.0 (0.8, 1.2) | 1.2 (0.6, 1.7 |
| Peru | 1990-2007 | 0.7 (0.4, 1.0) | 2007-2010 | 4.9 (-3.4, 13.8) | 2010-2019 | -0.2 (-1.0, 0.5) | 0.8 (0, 1.7) |
| Antigua and Barbuda | 1990-1994 | 4.2 (1.9, 6.5) | 1994-1999 | -2.1 (-4.3, 0) | 1999-2019 | 2.0 (1.8, 2.2) | 1.6 (1.1, 2.0) |
| Bahamas | 1990-1995 | -2.2 (-3.4, -1.0) | 1995-2013 | 0.7 (0.5, 0.9) | 2013-2019 | 0.1 (-0.8, 1.0) | 0.1 (-0.2, 0.3) |
| Barbados | 1990-2000 | 0.8 (0.6, 1.0) | 2000-2004 | 3.6 (2.3, 4.8) | 2004-2019 | 0.2 (0.1, 0.3) | 0.9 (0.7, 1.1) |
| Belize | 1990-1992 | 0.9 (-3.8, 5.9) | 1992-2000 | 5.6 (4.9, 6.3) | 2000-2019 | 0.2 (0.1, 0.3) | 1.7 (1.3, 2.1) |
| Bermuda | 1990-1998 | -2.6 (-2.8, -2.4) | 1998-2005 | -1.9 (-2.3, -1.5) | 2005-2019 | 0.1 (0, 0.2) | -1.1 (-1.3, -1.0) |
| Cuba | 1990-2000 | -1.3 (-1.7, -0.8) | 2000-2005 | 2.8 (1.0, 4.7) | 2005-2019 | 2.0 (1.7, 2.3) | 1.0 (0.7, 1.4) |
| Dominica | 1990-1998 | -0.1 (-0.5, 0.3) | 1998-2016 | 1.2 (1.1, 1.4) | 2016-2019 | 0.1 (-1.7, 1.9) | 0.8 (0.5, 1.0) |
| Dominican Republic | 1990-2000 | -0.2 (-0.8, 0.4) | 2000-2005 | 7.5 (4.9, 10.2) | 2005-2019 | 4.6 (4.2, 5.0) | 3.4 (2.9, 3.9) |
| Grenada | 1990-1994 | 3.5 (1.7, 5.3) | 1994-1998 | -0.5 (-3.2, 2.3) | 1998-2019 | 2.0 (1.9, 2.2) | 1.9 (1.4, 2.3) |
| Guyana | 1990-2003 | 1.9 (1.6, 2.3) | 2003-2009 | 0 (-1.5, 1.4) | 2009-2019 | 1.8 (1.3, 2.3) | 1.5 (1.1, 1.8) |
| Haiti | 1990-1998 | -1.4 (-1.5, -1.2) | 1998-2007 | 2.0 (1.8, 2.2) | 2007-2019 | 1.1 (0.9, 1.2) | 0.7 (0.6. 0.8) |
| Jamaica | 1990-1995 | 2.1 (-1.1, 5.4) | 1995-2000 | 5.3 (0.7, 10.1) | 2000-2019 | 2.0 (1.5, 2.4) | 2.6 (1.6, 3.5) |
| Puerto Rico | 1990-1999 | -0.4 (-0.9, 0) | 1999-2009 | 0.5 (0, 1.0) | 2009-2019 | -0.1 (-0.5, 0.3) | 0 (-0.2, 0.2) |
| Saint Kitts and Nevis | 1990-1992 | -5.9 (-11.4, 0) | 1992-2011 | -0.4 (-0.6, -0.2) | 2011-2019 | 1.8 (1.2, 2.5) | -0.2 (-0.6, 0.2) |
| Saint Lucia | 1990-1993 | 2.7 (0.2, 5.2) | 1993-2011 | -0.7 (-0.9, -0.6) | 2011-2019 | 3.2 (2.6, 3.7) | 0.7 (0.4, 1.0) |
| Saint Vincent and the Grenadines | 1990-2007 | 0.8 (0.6, 0.9) | 2007-2014 | 4.1 (3.4, 4.8) | 2014-2019 | 0.9 (0, 1.9) | 1.6 (1.4, 1.8) |
| Suriname | 1990-1993 | 1.8 (-3.1, 6.9) | 1993-1996 | -6.4 (-15.2, 3.3) | 1996-2019 | 2.2 (1.9, 2.4) | 1.2 (0.1, 2.3) |
| Trinidad and Tobago | 1990-2002 | -1.2 (-1.5, -0.9) | 2002-2005 | 2.6 (-2.6, 8.1) | 2005-2019 | 0.7 (0.4, 0.9) | 0.1 (-0.4, 0.6) |
| United States Virgin Islands | 1990-2005 | 1.3 (1.0, 1.5) | 2005-2009 | 6.3 (3.3, 9.4) | 2009-2019 | 0.1 (-0.4, 0.5) | 1.5 (1.1, 2.0) |
| Colombia | 1990-2003 | 0.5 (0.2, 0.7) | 2003-2006 | -1.1 (-5.6, 3.7) | 2006-2019 | 0.6 (0.4, 0.9) | 0.4 (-0.1, 0.9) |
| Costa Rica | 1990-1995 | 2.6 (0.9, 4.3) | 1995-2008 | 0.5 (0.1, 1.0) | 2008-2019 | 1.8 (1.3, 2.3) | 1.4 (1.0, 1.8) |
| El Salvador | 1990-2004 | 3.3 (3.0, 3.6) | 2004-2012 | -0.4 (-1.2, 0.4) | 2012-2019 | 1.9 (1.1, 2.7) | 1.9 (1.6, 2.2) |
| Guatemala | 1990-1994 | 6.8 (4.8, 8.8) | 1994-2001 | -0.5 (-1.5, 0.5) | 2001-2019 | 1.3 (1.2, 1.5) | 1.6 (1.3, 2.0) |
| Honduras | 1990-1998 | 5.8 (4.9, 6.8) | 1998-2015 | 2.8 (2.4, 3.1) | 2015-2019 | -0.4 (-3.0, 2.3) | 3.2 (2.7, 3.6) |
| Mexico | 1990-1998 | 1.1 (0.7, 1.4) | 1998-2011 | 0.6 (0.5, 0.8) | 2011-2019 | 1.5 (1.2, 1.8) | 1.0 (0.8, 1.1) |
| Nicaragua | 1990-1996 | 6.9 (4.9, 8.9) | 1996-2004 | 3.6 (2.1, 5.1) | 2004-2019 | 1.1 (0.7, 1.6) | 3.0 (2.4, 3.6) |
| Panama | 1990-2001 | 2.8 (2.6, 3.1) | 2001-2013 | 3.3 (3.1, 3.6) | 2013-2019 | 2.0 (1.3, 2.7) | 2.9 (2.7, 3.1) |
| Venezuela (Bolivarian Republic of) | 1990-2005 | -1.3 (-1.6, -1.0) | 2005-2008 | 3.8 (-3.5, 11.6) | 2008-2019 | 1.1 (l.6,1.6) | 0.1 (-0.6, 0.9) |
| Brazil | 1990-2004 | 2.0 (1.9, 2.0) | 2004-2013 | 1.0 (0.8, 1.2) | 2013-2019 | 0 (-0.3, 0.3) | 1.2 (1.2, 1.3) |
| Paraguay | 1990-2000 | 3.0 (2.6, 3.3) | 2000-2014 | 1.1 (0.9,1.4) | 2014-2019 | 2.0 (1.0, 3.0) | 1.9 (1.7, 2.1) |
| Afghanistan | 1990-2002 | -2.2 (-2.5, -1.9) | 2002-2014 | 5.4 (5.1, 5.8) | 2014-2019 | 2.1 (1.1, 3.2) | 1.6 (1.4, 1.9) |
| Algeria | 1990-2001 | 1.2 (1.1, 1.3) | 2001-2010 | 2.3 (2.2, 2.4) | 2010-2019 | 1.0 (0.9, 1.1) | 1.5 (1.4, 1.5) |
| Bahrain | 1990-2007 | 0.5 (0.1, 1.0) | 2007-2015 | -2.5 (-4.1, -0.8) | 2015-2019 | 1.9 (-2.1, 6.1) | -0.1 (-0.8, 0.6) |
| Egypt | 1990-1999 | 2.2 (1.8, 2.6) | 1999-2009 | 3.8 (3.4, 4.2) | 2009-2019 | 0.8 (0.4, 1.1) | 2.2 (2.0, 2.4) |
| Iran (Islamic Republic of) | 1990-1998 | 0.6 (0.2, 1.0) | 1998-2011 | 1.5 (1.3, 1.7) | 2011-2019 | 2.6 (2.3, 3.0) | 1.6 (1.4, 1.7) |
| Iraq | 1990-2004 | -0.8 (-1.0, -0.6) | 2004-2014 | 3.6 (3.2, 4.0) | 2014-2019 | 1.2 (0.2, 2.2) | 1.0 (0.8, 1.3) |
| Jordan | 1990-2002 | 0.8 (0.6, 1.1) | 2002-2005 | 3.2 (-1.0, 7.6) | 2005-2019 | 0.4 (0.2, 0.6) | 0.9 (0.5, 1.3) |
| Kuwait | 1990-1992 | -12.3 (-21.5, -2.0) | 1992 -2008 | 3.3 (2.8, 3.8) | 2008-2019 | -0.6 (-1.4, 0.1) | 0.6 (-0.2, 1.5) |
| Lebanon | 1990-1998 | 0.2 (0, 0.4) | 1998-2013 | 2.8 (2.7, 2.8) | 2013-2019 | 0.9 (0.6, 1.1) | 1.7 (1.6, 1.7) |
| Libya | 1990-2006 | 1.2 (1.0, 1.4) | 2006-2009 | 5.2 (-0.1, 10.9) | 2009-2019 | 0 (-0.4, 0.4) | 1.2 (0.7, 1.7) |
| Morocco | 1990-1993 | 2.8 (1.2, 4.4) | 1993-2003 | 1.2 (1.0, 1.5) | 2003-2019 | 3.1 (3.0, 3.3) | 2.4 (2.3, 2.6) |
| Oman | 1990-2009 | 5.8 (5.7, 5.9) | 2009-2017 | 0.3 (-0.1, 0.8) | 2017-1019 | -3.0 (-6.3, 0.3) | 3.6 (3.4, 3.9) |
| Palestine | 1990-1995 | 0.8 (-0.1, 1.8) | 1995-2007 | -0.4 (-0.7, -0.1) | 2007-2019 | 3.3 (3.1, 3.6) | 1.3 (1.1, 1.6) |
| Qatar | 1990-1995 | -0.1 (-2.5, 2.3) | 1995-2009 | 2.4 (1.9, 3.0) | 2009-2019 | 0.2 (-0.6, 1.1) | 1.2 (0.7, 1.8) |
| Saudi Arabia | 1990-1996 | 6.5 (6.1, 6.9) | 1996-2000 | 4.0 (2.9, 5.1) | 2000-2019 | 0.8 (0.7, 0.9) | 2.4 (2.2, 2.6) |
| Sudan | 1990-2001 | 1.6 (1.3, 1.8) | 2001-2016 | 4.5 (4.3, 4.6) | 2016-2019 | 2.4 (0.8, 4.1) | 3.1 (2.9, 3.3) |
| Syrian Arab Republic | 1990-1999 | 2.6 (2.1, 3.0) | 199-2004 | -2.6 (-4.1, -1.1) | 2004-2019 | 0.8 (0.6, 1.0) | 0.8 (0.5, 1.1) |
| Tunisia | 1990-2006 | 1.4 (1.3, 1.6) | 2006-2013 | 2.2 (1.6, 2.9) | 2013-2019 | 0.9 (0.3, 1.6) | 1.5 (1.3, 1.7) |
| Turkey | 1990-2004 | -1.2 (-1.5, -0.9) | 2004-2011 | 3.2 (2.0, 4.3) | 2011-2019 | 1.1 (0.4, 1.8) | 0.5 (0.1, 0.8) |
| United Arab Emirates | 1990-2001 | 2.8 (2.1, 3.4) | 2001-2006 | 5.7 (2.7, 8.7) | 2006-2019 | -1.4 (-1.9, -0.9) | 1.4 (0.8, 1.9) |
| Yemen | 1990-1999 | 0.8 (0.6, 1.0) | 1999-2014 | 3.9 (3.8, 4.0) | 2014-2019 | -0.3 (-0.8, 0.1) | 2.2 (2.1, 2.3) |
| Bangladesh | 1990-1998 | 2.0 (1.3, 2.7) | 1998-2007 | 5.4 (4.7, 6.2) | 2007-2019 | 2.9 (2.5, 3.3) | 3.4 (3.1, 3.8) |
| Bhutan | 1990-1996 | 3.0 (2.7, 3.2) | 1996-2004 | 4.6 (4.4, 4.8) | 2004-2019 | 2.8 (2.7, 2.8) | 3.3 (3.3, 3.4) |
| India | 1990-2005 | 4.6 (4.3, 4.9) | 2005-2010 | 0.6 (-1.4, 2.7) | 2010-2019 | 4.2 (3.6, 4.8) | 3.8 (3.4, 4.2) |
| Nepal | 1990-1998 | 2.4 (1.0, 2.7) | 1998-2016 | 6.2 (6.1, 6.3) | 2016-2019 | 3.3 (1.8, 4.8) | 4.8 (4.6, 5.0) |
| Pakistan | 1990-1996 | 4.5 (4.1, 4.8) | 1996-2005 | 6.6 (6.4, 6.8) | 2005-2019 | 1.7 (1.6, 1.8) | 3.8 (3.7, 3.9) |
| China | 1990-2001 | 2.2 (2.1, 2.4) | 2001-2016 | 0 (-0.1, 0.1) | 2016-2019 | 3.5 (2.5, 4.5) | 1.2 (1.1, 1.3) |
| Democratic People's Republic of Korea | 1990-1997 | 0.2 (0, 0.4) | 1997-2003 | -0.7 (-1.0, -0.4) | 2003-2019 | -0.1 (-0.1, 0) | -0.1 (-0.2, 0) |
| Taiwan (Province of China) | 1990-1996 | 4.3 (3.4, 5.2) | 1996-2006 | 2.6 (2.1, 3.1) | 2006-2019 | 0.3 (0.1, 0.6) | 1.9 (1.7, 2.2) |
| American Samoa | 1990-2006 | 1.6 (1.4, 1.7) | 2006-2009 | -0.9 (-5.3, 3.7) | 2009-2019 | 1.2 (0.8, 1.5) | 1.2 (0.7, 1.6) |
| Cook Islands | 1990-2006 | 0.1 (0, 0.1) | 2006-2009 | -0.2 (-2.0, 1.5) | 2009-2019 | 1.1 (1.0, 1.3) | 0.4 (0.2, 0.6) |
| Fiji | 1990-2005 | 1.6 (1.5, 1.7) | 2005-2008 | 2.1 (-0.6, 4.9) | 2008-2029 | 0.9 (0.7, 1.1) | 1.4 (1.1, 1.7) |
| Guam | 1990-1996 | -2.8 (-4.1, -1.5) | 1996-2010 | 0.9 (0.5, 1.3) | 2010-2019 | 2.3 (1.5, 3.0) | 0.5 (0.1, 0.9) |
| Kiribati | 1990-2005 | 1.4 (1.3, 1.4) | 2005-2013 | -2.5 (-2.8, -2.3) | 2013-2019 | 1.0 (0.7, 1.3) | 0.2 (0.1, 0.3) |
| Marshall Islands | 1990-2004 | 2.8 (2.6, 3.0) | 2004-2011 | -0.6 (-1.2, 0.1) | 2011-2019 | 1.1 (0.6, 1.5) | 1.5 (1.3, 1.7) |
| Micronesia (Federated States of) | 1990-2004 | 2.2 (2.0, 2.3) | 2004-2013 | -0.3 (-0.6, 0) | 2013-2019 | 0.8 (0.3, 1.3) | 1.1 (1.0, 1.2) |
| Nauru | 1990-2002 | 0.3 (0.2, 0.5) | 2002-2011 | -1.0 (-1.2, -0.7) | 2011-2019 | 2.4 (2.1, 2.6) | 0.5 (0.4, 0.6) |
| Niue | 1990-1993 | 3.0 (2.0, 4.0) | 1993-2005 | 1.3 (1.2, 1.4) | 2005-2019 | 0.2 (0.2, 0.3) | 1.0 (0.8, 1.1) |
| Northern Mariana Islands | 1990-2000 | 0.1 (-0.3, 0.5) | 2000-2004 | 2.8 (0.2, 5.6) | 2004-2019 | 0.1 (-0.2, 0.3) | 0.5 (0.1, 0.8) |
| Palau | 1990-2004 | 1.3 (1.2, 1.4) | 2004-2011 | -0.4 (-0.7, -0.1) | 2011-2019 | 0.8 (0.6, 1.0) | 0.7 (0.6, 0.8) |
| Papua New Guinea | 1990-2003 | 2.1 (1.9, 2.2) | 2003-2013 | -0.7 (-1.0, -0.4) | 2013-2019 | 1.2 (0.6, 1.8) | 0.9 (0.7, 1.1) |
| Samoa | 1990-2003 | 0.4 (0.3, 0.5) | 2003-2014 | -0.9 (-1.0, -0.8) | 2014-2019 | 1.2 (0.8, 1.6) | 0 (-0.1, 0.1) |
| Solomon Islands | 1990-2004 | 4.7 (4.2, 5.2) | 2004-2016 | 0.5 (-0.2, 1.2) | 2016-2019 | 9.2 (3.7, 15.1) | 3.4 (2.7, 4.0) |
| Tokelau | 1990-2004 | 1.9 (1.8, 2.0) | 2004-2010 | 0 (-0.4, 0.5) | 2010-2019 | 1.0 (0.8, 1.2) | 1.2 (1.1, 1.3) |
| Tonga | 1990-2002 | 2.9 (2.7, 3.0) | 2002-2010 | -1.6 (-1.9, -1.3) | 2010-2019 | 0.5 (0.2, 0.7) | 0.9 (0.7, 1.0) |
| Tuvalu | 1990-2001 | 2.6 (2.4, 2.9) | 2001-2013 | -0.3 (-0.6, -0.1) | 2013-2019 | 1.1 (0.5, 1.7) | 1.1 (0.9, 1.2) |
| Vanuatu | 1990-1997 | 3.2 (2.2, 4.1) | 1997-2003 | 0.1 (-1.5, 1.7) | 2003-2019 | 1.7 (1.3, 2.0) | 1.7 (1.3, 2.1) |
| Cambodia | 1990-2002 | 1.9 (1.9, 2.0) | 2002-2012 | 4.6 (4.5, 4.7) | 2012-2019 | 3.5 (3.3, 3.7) | 3.2 (3.2, 3.3) |
| Indonesia | 1990-2000 | 3.7 (3.6, 3.8) | 2000-2012 | 5.8 (5.6, 5.9) | 2010-2019 | 3.5 (3.4, 3.6) | 4.4 (4.3, 4.4) |
| Lao People's Democratic Republic | 1990-2001 | 2.3 (2.3, 2.4) | 2001-2009 | 3.8 (3.7, 3.9) | 2009-2019 | 3.1 (2.0, 3.2) | 3.0 (3.0 ,3.0) |
| Malaysia | 1990-1999 | 3.5 (3.1, 3.9) | 1999-2014 | 1.8 (1.6, 2.0) | 2014-2019 | 3.1 (2.2, 4.0) | 2.5 (2.3, 2.7) |
| Maldives | 1990-1997 | 2.8 (2.1, 3.4) | 1997-2004 | 0.6 (-0.2, 1.5) | 2014-2019 | 2.9 (2.6, 3.1) | 2.3 (2.0, 2.6) |
| Mauritius | 1990-1999 | 2.3 (1.8, 2.9) | 1999-2003 | 3.8 (1.0. 6.8) | 2003-2019 | 1.7 (1.5, 1.9) | 2.2 (1.8, 2.6) |
| Myanmar | 1990-2001 | 2.2 (1.9, 2.4) | 2001-2014 | 4.7 (4.4, 4.9) | 2014-2019 | 1.3 (0.5, 2.2) | 3.1 (2.9, 3.3) |
| Philippines | 1990-1997 | 3.6 (2.9, 4.3) | 1997-2008 | -1.2 (-1.6, -0.7) | 2008-2019 | 3.6 (3.2, 3.9) | 1.8 (1.5, 2.0) |
| Seychelles | 1990-2004 | 2.5 (2.3, 2.6) | 2004-2011 | -0.9 (-1.6, -0.3) | 2011-2019 | 2.0 (1.6, 2.5) | 1.5 (1.3, 1.7) |
| Sri Lanka | 1990-1993 | 0.2 (-2.4, 2.8) | 1993-1997 | 7.2 (4.5, 9.9) | 1997-2019 | 1.8 (1.7, 1.9) | 2.3 (1.9, 2.8) |
| Thailand | 1990-2000 | 5.2 (4.9, 5.6) | 2000-2010 | 2.2 (1.8, 2.6) | 2010-2019 | 1.6 (1.3, 2.0) | 3.1 (2.9, 3.3) |
| Timor-Leste | 1990-2004 | -1.2 (-1.5, -0.9) | 2004-2015 | 6.5 (6.0, 7.1) | 2015-2019 | 1.2 (-0.9, 3.4) | 2.0 (1.6, 2.4) |
| Viet Nam | 1990-2000 | 2.7 (2.4, 2.9) | 2000-2010 | 8.1 (7.8, 8.4) | 2010-2019 | 3.6 (3.3, 3.9) | 4.8 (4.7, 5.0) |
| Angola | 1990-1996 | 1.7 (1.1, 2.3) | 1996-2007 | 2.7 (2.5, 3.0) | 2007-2019 | 5.0 (4.8, 5.2) | 3.4 (3.3, 3.6) |
| Central African Republic | 1990-2002 | 1.8 (1.6, 1.9) | 2002-2014 | 0.5 (0.4, 0.6) | 2014-2019 | -1.2 (-1.7, -0.7) | 0.7 (0.6, 0.8) |
| Congo | 1990-1997 | 2.9 (2.4, 3.3) | 1997-2003 | -1.1 (-1.8, -0.4) | 2003-2019 | 1.5 (1.4, 1.6) | 1.3 (1.1, 1.5) |
| Democratic Republic of the Congo | 1990-1995 | -0.6 (-1.4, 0.2) | 1995-2009 | -3.7 (-3.9, -3.5) | 2009-2019 | 4.0 (3.7, 4.3) | -0.6 (-0.8, -0.4) |
| Equatorial Guinea | 1990-1995 | 2.1 (0.6, 3.6) | 11.1 (10.7, 11.6) | 3.5 (2.0. 4.0) | 2008-2019 | 3.5 (3.0, 4.0) | 6.6 (6.2, 6.9) |
| Gabon | 1990-2001 | 4.8 (4.5, 5.0) | 2001-2014 | 0.8 (0.6, 1.0) | 2014-2019 | 2.1 (1.3, 3.0) | 2.5 (2.3, 2.7) |
| Burundi | 1990-1995 | -0.6 (-1.4, 0.2) | 1995-2009 | -3.7 (-3.9, -3.5) | 2009-2019 | 4.0 (3.7, 4.3) | -0.6 (-0.8, -0.4) |
| Comoros | 1990-1996 | 3.3 (2.5, 4.2) | 1996-2006 | 0 (-0.5, 0.4) | 2006-2019 | 1.7 (1.4, 1.9) | 1.4 (1.2, 1.7) |
| Djibouti | 1990-1997 | 2.2 (1.7, 2.7) | 1997-2006 | 5.9 (5.5, 6.4) | 2006-2019 | 3.1 (2.9, 3.3) | 3.8 (3.6, 4.4) |
| Eritrea | 1990-2003 | 6.6 (6.3, 6.9) | 2003-2011 | 0.5 (-0.3, 1.2) | 2011-2019 | 2.5 (1.9, 3.2) | 3.7 (3.5, 4.0) |
| Ethiopia | 1990-2001 | -0.5 (-0.6, -0.4) | 2001-2008 | 2.0 (1.7, 2.3) | 2008-2019 | 5.1 (5.0, 5.3) | 2.2 (2.1, 2.3) |
| Kenya | 1990-2000 | 2.1 (2.0, 2.2) | 2000-2011 | 5.6 (5.5, 5.7) | 2011-2019 | 1.9 (1.7, 2.1) | 3.4 (3.3, 3.4) |
| Madagascar | 1990-1994 | -2.3 (-4.4, -0.1) | 1994-2012 | 2.3 (2.2, 2.7) | 2012-2019 | 3.2 (2.2, 4.2) | 1.9 (1.5, 2.3) |
| Malawi | 1990-1995 | 0.9 (0.3, 1.6) | 1995-2004 | 7.7 (7.4, 8.1) | 2004-2019 | 1.6 (1.5, 1.7) | 3.4 (3.2, 3.5) |
| Mozambique | 1990-1996 | 1.3 (0.8, 1.8) | 1996-2014 | 6.2 (6.0-6.3) | 2014-2019 | 2.6 (2.0, 3.3) | 4.5 (4.4, 4.7) |
| Rwanda | 1990-1996 | 0.4 (-0.5, 1.2) | 1996-2006 | -1.1 (-1.6, -0.7) | 2006-2019 | 3.9 (3.6, 4.2) | 1.4 (1.2, 1.7) |
| Somalia | 1990-1994 | -1.7 (-2.2, -1.2) | 1994-2014 | 0.4 (0.3, 0.4) | 2014-2019 | -0.3 (-0.7, 0) | 0 (-0.1, 0) |
| South Sudan | 1990-1996 | 0.2 (-0.1, 0.5) | 1996-2012 | 1.6 (1.5, 1.7) | 2012-2019 | 1.3 (1.0, 1.5) | 1.2 (1.1, 1.3) |
| Uganda | 1990-1996 | 2.3 (1.5, 3.2) | 1996-2005 | 6.2 (5.7, 6.8) | 2005-2019 | 4.4 (4.2, 4.7) | 4.5 (4.3, 4.8) |
| United Republic of Tanzania | 1990-2001 | 0.2 (0, 0.4) | 2001-2016 | 2.4 (2.3, 2.5) | 2016-2019 | 4.3 (2.9, 5.7) | 1.7 (1.6, 1.9) |
| Zambia | 1990-1999 | 2.1 (2.0, 2.2) | 1999-2008 | 0 (-0.1, 0.2) | 2008-2019 | 3.6 (3.5, 3.7) | 2.0 (1.9, 2.1) |
| Botswana | 1990-2002 | 5.9 (5.6, 6.1) | 2002-2006 | -0.6 (-2.6, 1.5) | 2006-2019 | 2.2 (2.0, 2.5) | 3.3 (3.0, 3.6) |
| Eswatini | 1990-1995 | 3.3 (2.3, 4.3) | 1995-2003 | 8.3 (7.7, 8.9) | 2003-2019 | -1.5 (-1.7, -1.4) | 1.9 (1.7, 2,2) |
| Lesotho | 1990-1996 | 3.6 (2.5, 4.7) | 1996-2004 | 8.2 (7.2, 9.1) | 2004-2019 | 2.5 (2.2, 2.8) | 4.3 (3.9, 4.6) |
| Namibia | 1990-2004 | 3.8 (3.5, 4.1) | 2004-2008 | -0.6 (-3.5, 2.5) | 2008-2019 | 3.0 (2.5, 3.4) | 2.9 (2.4, 3.3) |
| South Africa | 1990-1995 | 3.9 (2.1, 5.6) | 1995-1998 | 8.2 (0.3, 16.7) | 1998-2019 | -0.6 (-0.8, -0.4) | 1.1 (0.2, 1.9) |
| Zimbabwe | 1990-2004 | 3.1 (2.8, 3.3) | 2004-2012 | -0.8 (-1.4, -0.1) | 2012-2019 | 1.8 (1.2, 2.4) | 1.7 (1.5, 1.9) |
| Benin | 1990-1996 | 3.9 (3.7, 4.1) | 1996-2010 | 3.2 (3.1, 3.3) | 2010-1019 | 0.9 (0.8, 1.0) | 2.6 (2.6, 2.7) |
| Burkina Faso | 1990-2003 | 3.4 (3.3, 3.5) | 2003-2017 | 4.0 (3.9, 4.2) | 2017-2019 | 1.9 (-0.3, 4.1) | 3.6 (3.4, 3.8) |
| Cabo Verde | 1990-2000 | 8.2 (7.3, 9.1) | 2000-2015 | 2.7 (2.2, 3.3) | 2015-2019 | 8.9 (5.1, 12.8) | 5.4 (4.8, 6.1) |
| Cameroon | 1990-2003 | 1.3 (1.2, 1.4) | 2003-2013 | 2.3 (2.1, 2.5) | 2013-2019 | 1.2 (0.9, 1.5) | 1.6 (1.5, 1.7) |
| Chad | 1990-1996 | 4.0 (3.4, 4.7) | 1996-2015 | 3.0 (2.9, 3.1) | 2015-2019 | 2.0 (0.9, 3.1) | 3.1 (2.9, 3.3) |
| Côte d'Ivoire | 1990-1995 | 3.3 (2.9, 3.7) | 1995-2008 | 0.5 (0.4, 0.6) | 2008-2019 | 1.5 (1.4, 1.7) | 1.4 (1.3, 1.5) |
| Gambia | 1990-2007 | 3.9 (3.5, 4.2) | 2007-2012 | 0.2 (-2.6, 3.1) | 2012-2019 | 3.7 (2.5, 5.0) | 3.2 (2.6, 3.8) |
| Ghana | 1990-1999 | 6.3 (6.1, 6.5) | 1999-2011 | 3.7 (3.5, 3.9) | 2011-2019 | 3.2 (2.9, 3.5) | 4.4 (4.2, 4.5) |
| Guinea | 1990-1998 | 2.2 (2.1, 2.3) | 1998-2014 | 2.0 (1.9, 2.0) | 2014-2019 | 1.3 (1.2, 1.5) | 1.9 (1.9, 2.0) |
| Guinea-Bissau | 1990-1996 | 2.9 (2.7, 3.1) | 1996-2010 | 1.9 (1.8, 1.9) | 2010-2019 | 1.4 (1.4, 1.5) | 1.9 (1.9, 2.0) |
| Liberia | 1990-2000 | -4.1 (-4.3, -3.9) | 2000-2012 | 6.2 (6.0, 6.5) | 2012-2019 | 1.0 (0.6, 1.4) | 1.2 (1.2, 1.4) |
| Mali | 1990-1996 | 4.0 (3.5, 4.6) | 1996-2011 | 2.6 (2.4, 2.7) | 2011-2019 | 1.9 (1.5, 2.3) | 2.7 (2.5, 2.8) |
| Mauritania | 1990-2003 | 0.8 (0.6, 1.0) | 2003-2013 | 1.7 (1.3, 2.0) | 2013-2019 | 0.7 (0.2, 1.3) | 1.1 (0.9, 1.2) |
| Niger | 1990-1997 | 1.1 (0.6, 1.5) | 1997-2002 | 3.1 (2.0, 4.1) | 2002-2019 | 1.7 (1.6, 1.8) | 1.8 (1.6, 2.0) |
| Nigeria | 1990-2001 | 2.5 (2.3, 2.8) | 2001-2014 | 3.6 (3.4, 3.9) | 2014-2019 | 2.3 (1.4, 3.1) | 3.0 (2.8, 3.2) |
| Sao Tome and Principe | 1990-2002 | 3.4 (3.2, 3.6) | 2002-2005 | 2.4 (-0.8, 5.6) | 2005-2019 | 3.4 (3.2, 3.5) | 3.3 (3.0, 3.6) |
| Senegal | 1990-1999 | 0 (-0.5, 0.4) | 1999-2002 | 5.9 (1.1, 10.9) | 2002-2019 | 2.6 (2.4, 2.7) | 2.1 (1.6, 2.6) |
| Sierra Leone | 1990-2002 | 0.9 (0.8, 1.1) | 2002-2017 | 3.5 (3.4, 3.6) | 2017-2019 | 2.0 (0.1, 4.0) | 2.3 (2.2, 2.5) |
| Togo | 1990-1998 | 3.2 (3.0, 3.5) | 1998-2006 | 1.7 (1.3, 2.0) | 2006-2019 | 2.3 (2.2, 2.5) | 2.4 (2.3, 2.5) |

Abbreviations: AAPC, average annual percentage change; APC, annual percentage change; ASMR, age-standardized mortality rate.
